# Sulfide Oxidation to Sulfone Using Sodium Chlorite and Hydrochloric Acid in Organic Solvents

**DOI:** 10.3390/molecules30091912

**Published:** 2025-04-25

**Authors:** Yuki Itabashi, Shuto Ogata, Tsuyoshi Inoue, Haruyasu Asahara, Kei Ohkubo

**Affiliations:** 1 Institute for Open and Transdisciplinary Research Initiatives (OTRI), The University of Osaka, 1-6 Yamada-oka, Suita 565-0871, Osaka, Japan; 2 Graduate School of Pharmaceutical Sciences, The University of Osaka, 1-6 Yamada-oka, Suita 565-0871, Osaka, Japan

**Keywords:** sodium chlorite, chlorine dioxide, sulfur oxidation, sulfone, oxygenation, selective oxidation

## Abstract

Organosulfur compounds are appealing owing to the diverse oxidation states accessible by sulfur, allowing the precise adjustment of their properties. In this study, we report a practical oxidation method that converts sulfides to sulfones by generating chlorine dioxide in situ from sodium chlorite (NaClO_2_) and hydrochloric acid (HCl) in organic solvents. Diphenyl sulfide was effectively oxidized to diphenyl sulfone in yields of up to 96% under optimized conditions, with high selectivity in ethyl acetate and acetonitrile solvents. The method is compatible with a wide range of substrates, including various aryl, benzyl, and alkyl sulfides, although reactivity diminishes with sterically hindered or electron-rich substrates. This scalable and environmentally friendly process overcomes challenges associated with aqueous oxidants, such as substrate solubility and side reactions, providing a robust alternative for sulfone synthesis.

## 1. Introduction

Organosulfur compounds exhibit various oxidation states, enabling the precise adjustment of their physical and chemical characteristics. Owing to this adaptability, organosulfur frameworks have become essential in natural product synthesis, pharmaceuticals, polymer resins, and organic electronic materials [1,2,3]. Among sulfur oxidation processes, sulfone synthesis is particularly significant because of the broad applicability of sulfones as synthetic intermediates and functional materials [4]. Traditional oxidants, such as hydrogen peroxide (H_2_O_2_), *m*-chloroperoxybenzoic acid (*m*CPBA), and sodium hypochlorite (NaClO), are commonly utilized for this transformation [5,6,7,8,9,10,11,12,13]. However, the aqueous reaction conditions associated with H_2_O_2_ and NaClO often present challenges concerning substrate solubility, reaction control, and unwanted side reactions for substrates that react with water.

Sodium chlorite (NaClO_2_), an affordable oxidizing agent obtained easily through NaCl electrolysis, serves as an attractive alternative. Despite its insolubility in organic solvents, the reaction of NaClO_2_ with hydrochloric acid (HCl) produces chlorine dioxide (ClO_2_^•^) [14], a powerful and selective oxidizing agent that functions effectively in organic media [15,16]. While tetrabutylammonium chlorite (TBAClO_2_), as previously reported, is also effective, synthesizing this reagent is not strictly required for sulfone formation [16]. Instead, using commercially available and inexpensive NaClO_2_ in concert with HCl offers a more convenient and practical option. In this study, we report a practical, scalable, and environmentally friendly technique for the targeted oxidation of sulfides to sulfones using ClO_2_^•^ generated in situ. This method provides high selectivity, operational simplicity, and gentle reaction conditions.

## 2. Results and Discussion

**Figure 1:**
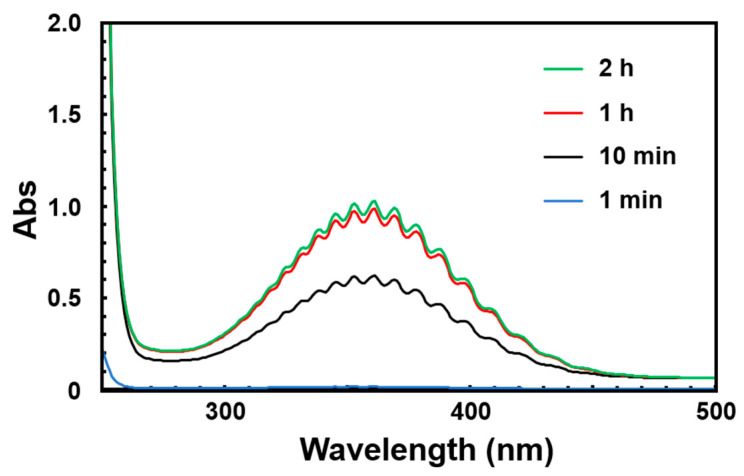
UV–Vis absorption spectra obtained during the reaction between NaClO_2_ (0.25 mM) and HCl (0.20 mM) in MeCN at 298 K.

ClO_2_^•^ is produced in an aqueous solution through the reaction between NaClO_2_ and HCl, resulting in a yellow solution (Equation (1)) [17,18]. Its ultraviolet–visible (UV–Vis) absorption spectrum exhibits a prominent peak at 358 nm [19,20], facilitating the spectroscopic confirmation of ClO_2_^•^ formation. This study investigated the potential production of ClO_2_^•^ in an organic solvent. Amounts of 57 mg of 0.50 mmol NaClO_2_, 400 μL of 0.40 mmol HCl in ethyl acetate (EtOAc), and 600 μL of acetonitrile (MeCN) were mixed and stirred at 25 °C for varying durations: 1 min, 10 min, 1 h, or 2 h. Subsequently, the supernatant was diluted twenty-fold, and UV–Vis spectroscopy was conducted at 25 °C (Figure 1). A slight ClO_2_^•^ absorption was observed at 1 min, while a distinct absorption at 358 nm appeared by 10 min, reaching maximum intensity within 1 h. The spectrum remained constant at 2 h, indicating completion of the reaction.

(1) $$5{NaClO}_{2}+4HCl⟶4{ClO}_{2}^{•}+2H_{2}O+5NaCl$$

**Figure 2:**
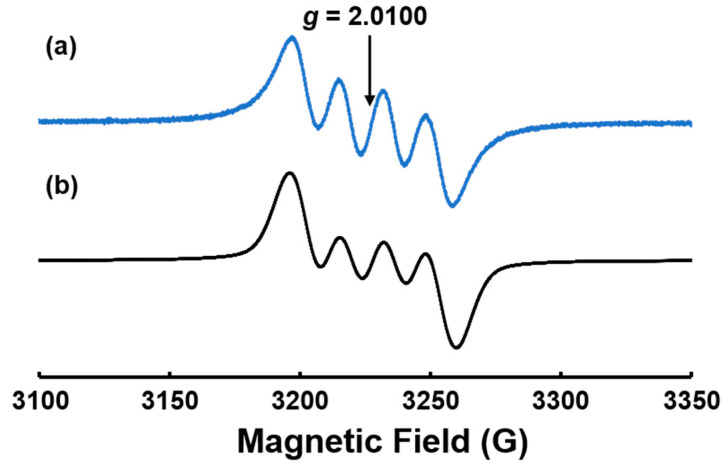
(**a**) ESR spectrum of a MeCN solution containing NaClO_2_ (0.25 mM) with HCl (0.20 mM) at 298 K. (**b**) The simulated spectrum with *a*(Cl) = 17.1 G and D*H*_msl_ = 14.7 G.

Electron spin resonance (ESR) measurements were conducted to confirm the formation of the radical species ClO_2_^•^ under the present conditions in EtOAc. ClO_2_^•^ was generated by combining a 1.0 M HCl solution in EtOAc with NaClO_2_ in EtOAc. Following the presence of solid residue, the supernatant was isolated and then diluted by a factor of 20. The ESR spectrum was obtained using the X-band and 298 K, revealing the characteristic isotropic signal at *g* = 2.010 with four hyperfine lines originating from the unpaired electron on the chlorine nucleus (*I* = 3/2 for ^35^Cl and ^37^Cl), as shown in Figure 2a. This confirms the production of ClO_2_^•^ under these experimental conditions [20,21,22]. The hyperfine coupling constant and maximum slope line widths were determined from the simulated spectrum (Figure 2b) to be *a*(Cl) = 16.7 G and Δ*H*_msl_ = 14.7 G, respectively. In EtOAc, the observed *g* value of ClO_2_^•^ differed from that reported in acetonitrile, emphasizing the impact of solvent polarity. In a more polar solvent such as acetonitrile, increased solvation and enhanced spin–orbit coupling cause a deviation of the *g* value from the free-electron value (*g* = 2.0023), resulting in a notably higher *g* value in acetonitrile compared to ethyl acetate.

Treatment of diphenyl sulfide (**1a**) with 5.0 equiv of NaClO_2_ and 4.0 equiv of HCl in a 0.040 M MeCN/HCl solution at room temperature for 1 h resulted in complete conversion, yielding diphenyl sulfone (**3a**) in 96% yield (Table 1, entry 1), without any detectable formation of diphenyl sulfoxide (**2a**). Although several HCl/organic solutions (e.g., 4 M HCl in dioxane, 3 M in MeOH, 2 M in Et_2_O, etc.) are commercially available, 1 M HCl in ethyl acetate was selected as the HCl source primarily due to safety considerations and chemical compatibility with chlorine dioxide. Dioxane and diethyl ether may react with chlorine dioxide to form explosive peroxides, posing a serious safety hazard, and are therefore unsuitable. In addition, MeOH reacts with ClO_2_^•^ as reported [17], rendering it incompatible with this oxidation system. Similar results were obtained using EtOAc (entry 2), whereas switching to toluene reduced the yield of **3a** to 30%, accompanied by a 45% yield of **2a** (entry 3). The use of water as the solvent led to incomplete conversion and a lower sulfone yield (entry 4). No reaction occurred in the absence of HCl, and **1a** remained unchanged (entry 5).

Increasing the reaction concentration to 0.20 M in EtOAc allowed for a reduction in NaClO_2_ and HCl loadings to 1.9 and 1.5 equiv, respectively, resulting in a yield of **3a** at 80% with **2a** at 14% (entry 6). Under these conditions, MeCN provided **3a** in a 95% yield, making it the optimal solvent (entry 7). Reducing the reaction time to 10 min yielded 85% of **3a** with 9% of **2a**, indicating incomplete conversion (entry 8). Further decreasing NaClO_2_ to 1.3 equivalents and HCl to 1.0 equivalents resulted in a 50% yield of **3a** and 43% of **2a** (entry 9).

**Figure 3:**
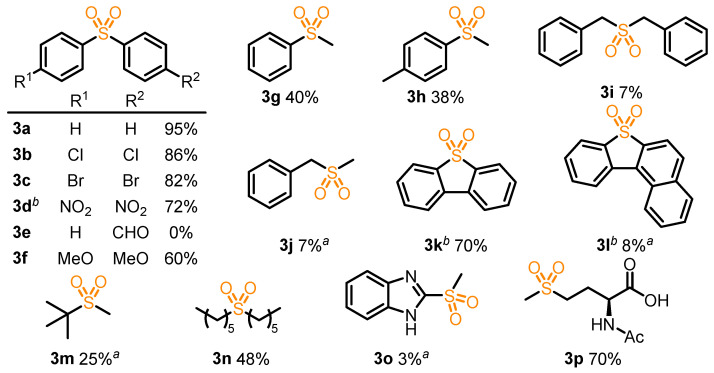
Substrate scope evaluation for sulfide oxygenation using NaClO_2_ in EtOAc. *^a^* Determined by ^1^H NMR analysis using 1,1,2,2-tetrachloroethane as an internal standard. *^b^* Reaction was conducted using 3.8 equiv of NaClO_2_ and 3.0 equiv of HCl in EtOAc.

Scaling the reaction to 0.50 mmol under the optimized conditions (Table 1, entry 7) provided **3a** in a 95% isolated yield (Figure 3). For comparison, the reaction using Bu_4_NClO_2_ under similar conditions has been reported to afford **3a** in 95% yield [15]. Evaluation of the substrate scope showed that sulfides with *p*-halogen substituents (**3b**, 86%; **3c**, 82%) were effectively oxidized. However, sulfides with *p*-nitro substitution required higher oxidant loading (6.3 equiv of NaClO_2_ and 5.0 equiv of HCl) to achieve complete conversion, affording **3d** in a 72% yield. Sulfides with formyl substitutions underwent simultaneous oxidation at both the sulfide and formyl groups, hindering the isolation of the desired sulfone. Electron-donating groups like *p*-methoxy led to moderate yields (**3f**, 60%), while methyl-substituted sulfides showed lower efficiency (**3g**, 40%; **3h**, 38%). Although other common oxidants often provide comparable yields for diphenyl sulfide, chlorine dioxide, being a radical species, preferentially reacts at positions that stabilize radical intermediates—such as benzylic or α-positions adjacent to heteroatoms—which can lead to side reactions and thus lower yields.

Benzyl-substituted sulfides exhibited reduced reactivity, with dibenzyl sulfide producing **3i** in an only 7% yield and benzyl methyl sulfide providing **3j** in a 7% yield. Dibenzothiophene was oxidized to **3k** in a 70% yield, while benzonaphthothiophene, with its extended π-system, showed lower conversion, resulting in **3l** in an only 8% yield. Dialkyl sulfides displayed varying reactivity: *tert*-butyl methyl sulfide provided **3m** in a 25% yield, and dipropyl sulfide provided **3n** in 48%. A benzimidazole-containing substrate underwent slow oxidation, yielding **3o** in 3%. Notably, an *N*-acetyl-protected methionine derivative was selectively converted to its sulfone in a 70% yield.

**Scheme 1:**
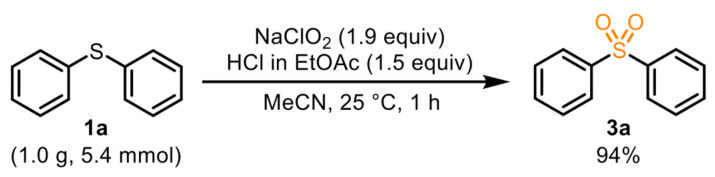
Gram-scale reaction.

The scalability of this method was demonstrated in gram-scale reactions, which proceeded smoothly, yielding **3a** in 94% from 1.00 g of **1a** (Scheme 1). These results emphasize the efficiency, wide substrate scope, and practical scalability of this ClO_2_-mediated sulfone synthesis, providing a promising alternative to traditional aqueous oxidation methods.

## 3. Materials and Methods

### 3.1. Materials

Sodium chlorite (technical grade, 80%) was purchased from Sigma-Aldrich (St. Louis, MO, USA). HCl in ethyl acetate (1 M) was purchased from TCI (Tokyo, Japan). All the other reagents were commercially available (reagent grade from TCI (Tokyo, Japan), FUJIFILM Wako Pure Chemical (Osaka, Japan), and Sigma-Aldrich (St. Louis, MO, USA)) and used as received. Nuclear magnetic resonance (NMR) spectra were recorded using a Bruker AVANCE NEO 400 spectrometer (Billerica, MA, USA), with chemical shifts calibrated using residual undeuterated solvent (CHCl_3_ at 7.26 ppm for ^1^H NMR, 77.16 ppm for ^13^C NMR; DMSO at 2.50 ppm for ^1^H NMR, 39.52 ppm for ^13^C NMR). The abbreviations for multiplicities are as follows: s (singlet), d (doublet), t (triplet), q (quartet), m (multiplet), and br (broad). High-resolution mass spectra were acquired with an AB SCEIX Triplet TOF 4600 mass spectrometer (Marsiling, Singapore).

### 3.2. UV–Vis Absorption Spectral Measurements

UV–Vis spectral measurements were conducted to determine the time taken to achieve the maximum generation of chlorine dioxide in the reaction between sodium chlorite and hydrogen chloride in acetonitrile. The spectra were captured using a JASCO V-750 UV–Vis Spectrophotometer. In a 9 mL screw-cap vial, sodium chlorite (57 mg, 0.50 mmol), hydrogen chloride in an EtOAc solution (400 μL, 0.40 mmol), and MeCN (600 μL) were combined. The mixture was then stirred for varying durations: 1 min, 10 min, 1 h, and 2 h at 25 °C. Subsequently, the supernatant was diluted 20-fold, and UV–Vis spectroscopy was conducted at the same temperature.

### 3.3. ESR Measurements

ClO_2_^•^ was produced by mixing NaClO_2_ (0.14 mg) and HCl (1.0 mg) in EtOAc (5.0 mL) at 298 K. The resulting solution was then transferred to a quartz ESR capillary tube with an internal diameter of 1.8 mm. ESR spectra were recorded using a JEOL X-band spectrometer (JES-X310) under nonsaturating microwave power conditions. The modulation magnitude was selected to enhance resolution and the signal-to-noise ratio (S/N) without altering the maximum slope linewidth (Δ*H*_msl_) of the ESR signals. The *g* values and hyperfine coupling (*hfc*) constants were calibrated using a Mn^2+^ marker and determined through computer simulation software (ver. 2.4.4) provided by JEOL Ltd. (Tokyo, Japan).

### 3.4. General Procedure for the Synthesis of ***3a***

To a 6 mL screw vial, diphenyl sulfide **1a** (93.1 mg, 0.50 mmol), sodium chlorite (105 mg, 0.95 mmol), MeCN (1.8 mL), and a solution of hydrogen chloride in ethyl acetate (750 μL, 0.75 mmol) were added. The mixture was stirred for 1 h at 25 °C. Subsequently, the mixture underwent purification by silica gel column chromatography using EtOAc as the eluent. After removal of the solvent under reduced pressure using a rotary evaporator, the desired product **3a** was obtained as a white solid (104 mg, 0.48 mmol, 95% yield).

### 3.5. Spectroscopic Data of Products

The NMR spectra of the known compounds matched those reported in the literature [23,24,25,26,27,28,29].

Diphenyl sulfone (**3a**). This compound was synthesized following the general procedure. A white solid was obtained (104 mg, 0.48 mmol, 95% yield). ^1^H NMR (400 MHz, CDCl_3_) δ (ppm): 8.04–7.88 (m, 4H), 7.65–7.43 (m, 6H). ^13^C NMR (100 MHz, CDCl_3_) δ (ppm): 144.4, 132.8, 126.2, 126.0. HRMS(MALDI): calcd for C_12_H_11_O_2_S [M + H]^+^: 219.0474, found: 219.0476.

Bis(4-chlorophenyl) sulfone (**3b**). This compound was synthesized following the general procedure. The resulting mixture underwent purification via silica gel column chromatography using a hexane/ethyl acetate ratio of 4:1. This process yielded a white solid (123 mg, 0.43 mmol, 86% yield). ^1^H NMR (400 MHz, CDCl_3_) δ (ppm): 7.86 (d, *J* = 8.8 Hz, 4H), 7.48 (d, *J* = 8.8 Hz, 4H). ^13^C NMR (100 MHz, CDCl_3_) δ (ppm): 140.3, 139.8, 129.8, 129.2. HRMS (ESI): calcd for C_12_H_8_O_2_NaSCl_2_ [M + Na]^+^:308.9517, found: 308.9514.

Bis(4-bromophenyl) sulfone (**3c**). This compound was synthesized following the general procedure. The resulting mixture underwent purification via silica gel column chromatography using a hexane/ethyl acetate ratio of 4:1. This process yielded a white solid (153 mg, 0.41 mmol, 82% yield). ^1^H NMR (400 MHz, CDCl_3_) δ (ppm): 7.78 (d, *J* = 8.8 Hz, 4H), 7.65 (d, *J* = 8.8 Hz, 4H). ^13^C NMR (100 MHz, CDCl_3_) δ (ppm): 140.3, 132.8, 129.3, 128.9. HRMS (ESI): calcd for C_12_H_8_O_2_NaSBr_2_ [M + Na]^+^: 396.8504, found: 396.8504.

Bis(4-nitrophenyl) sulfone (**3d**). This compound was synthesized following the general procedure using 3.8 equivalents of NaClO_2_ and 3.0 equivalents of hydrogen chloride. A yellow solid was obtained (111 mg, 0.36 mmol, 72% yield). ^1^H NMR (400 MHz, DMSO-*d_6_*) δ (ppm): 8.42 (d, *J* = 9.0 Hz, 4H), 8.30 (d, *J* = 9.0 Hz, 4H). ^13^C NMR (100 MHz, DMSO-*d_6_*) δ (ppm): 150.8, 144.8, 129.6, 125.2. HRMS (ESI): calcd for C_12_H_8_N_2_O_6_NaS [M + Na]^+^: 331.0001, found: 331.0003.

Bis(4-methoxyphenyl) sulfone (**3f**). This compound was synthesized following the general procedure. The resulting mixture underwent purification via silica gel column chromatography using a hexane ethyl acetate ratio of 4:1. This process yielded an orange solid (83.4 mg, 0.30 mmol, 60% yield). ^1^H NMR (400 MHz, CDCl_3_) δ (ppm): 7.83 (d, *J* = 9.0 Hz, 4H), 6.93 (d, *J* = 9.0 Hz, 4H), 3.81 (s, 6H). ^13^C NMR (100 MHz, CDCl_3_) δ (ppm): 163.1, 133.9, 129.5, 114.5, 55.7. HRMS (MALDI): calcd for C_14_H_15_O_4_S [M + H]^+^: 279.0686, found: 279.0684.

Methyl phenyl sulfone (**3g**). This compound was synthesized following the general procedure. The resulting mixture underwent purification via silica gel column chromatography using a hexane/ethyl acetate ratio of 4:1. This process yielded an orange solid (31.3 mg, 0.20 mmol, 40% yield). ^1^H NMR (400 MHz, CDCl_3_) δ (ppm): 7.99–7.87 (m, 2H), 7.71–7.61 (m, 1H), 7.60–7.50 (m, 2H), 3.04 (s, 3H). ^13^C NMR (100 MHz, CDCl_3_) δ (ppm): 140.5, 133.7, 129.4, 127.3, 44.5. HRMS (MALDI): calcd for C_7_H_8_O_2_NaS [M + Na]^+^: 179.0137, found: 179.0136.

Methyl 4-tolyl sulfone (**3h**). This compound was synthesized following the general procedure. The resulting mixture underwent purification via silica gel column chromatography using a hexane/ethyl acetate ratio of 4:1. This process yielded a white solid (32.3 mg, 0.19 mmol, 38% yield). ^1^H NMR (400 MHz, CDCl_3_) δ (ppm): 7.81 (d, *J* = 8.0 Hz, 2H), 7.35 (d, *J* = 8.0 Hz, 2H), 3.02 (s, 3H), 2.44 (s, 3H). ^13^C NMR (100 MHz, CDCl_3_) δ (ppm): 144.7, 137.7, 130.0, 127.4, 44.7, 21.7. HRMS (MALDI): calcd for C_8_H_10_O_2_NaS [M + H]^+^: 193.0294, found: 193.0296.

Dibenzyl sulfone (**3i**). This compound was synthesized following the general procedure on a 2.0 mmol scale. The resulting mixture underwent purification via silica gel column chromatography using a hexane/ethyl acetate ratio of 7:1. This process yielded a yellow solid (34.2 mg, 0.14 mmol, 7% yield). ^1^H NMR (400 MHz, CDCl_3_) δ (ppm): 7.51–7.29 (m, 10H), 4.13 (s, 4H). ^13^C NMR (100 MHz, CDCl_3_) δ (ppm): 130.9, 129.1, 129.1, 127.6, 58.0. HRMS (ESI): calcd for C_14_H_14_O_2_NaS [M + Na]^+^: 269.0608, found: 269.0606.

Benzyl methyl sulfone (**3j**). This compound was synthesized following the general procedure. The NMR yield was determined using 1,1,2,2-tetrachloroethane as an internal standard. ^1^H NMR (400 MHz, CDCl_3_) δ (ppm): 7.45–7.35 (m, 5H), 4.24 (s, 2H), 3.30 (s, 3H). ^13^C NMR (100 MHz, CDCl_3_) δ (ppm): 130.6, 129.2, 128.3, 128.2, 61.3, 39.1. HRMS (MALDI): C_8_H_10_O_2_NaS [M + Na]^+^: 193.0294, found: 193.0290.

Dibenzothiophene-5,5-dioxide (**3k**). This compound was synthesized following the general procedure using 3.8 equivalents of NaClO_2_ and 3.0 equivalents of hydrogen chloride. A white solid was obtained (97.2 mg, 0.45 mmol, 90% yield). ^1^H NMR (400 MHz, CDCl_3_) δ (ppm): 7.88–7.72 (m, 4H), 7.71–7.56 (m, 2H), 7.55–7.45 (m, 2H). ^13^C NMR (100 MHz, CDCl_3_) δ (ppm): 137.7, 134.0, 131.6, 130.4, 122.2, 121.7. HRMS (MALDI): calcd for C_12_H_9_O_2_S [M + H]^+^: 217.0318, found: 217.0317.

Benzo[b]naphtho[1,2-d]thiophene-11,11-dioxide (**3l**). This compound was synthesized following the general procedure using 3.8 equivalents of NaClO_2_ and 3.0 equivalents of hydrogen chloride. The NMR yield was determined using 1,1,2,2-tetrachloroethane as an internal standard. ^1^H NMR (400 MHz, CDCl_3_) δ (ppm): 8.79–8.75 (m, 1H), 8.50–8.47 (m, 1H), 8.44 (d, *J* = 7.6 Hz, 1H), 8.06–7.98 (m, 2H), 7.92 (d, *J* = 7.6 Hz, 1H), 7.86–7.79 (m, 2H), 7.74 (ddd, *J* = 1.2, 7.6, 7.6 Hz, 1H), 7.60 (ddd, *J* = 0.7, 7.6, 7.6 Hz, 1H). HRMS (ESI): calcd for C_16_H_10_O_2_NaS [M + Na]^+^: 289.0279, found: 289.0293.

*tert*-Butyl methyl sulfone (**3m**). This compound was synthesized following the general procedure. The NMR yield was determined using 1,1,2,2-tetrachloroethane as an internal standard. ^1^H NMR (400 MHz, CDCl_3_) δ (ppm): 2.81 (s, 3H), 1.42 (s, 9H).

Dihexyl sulfone (**3n**). This compound was synthesized following the general procedure on a 1.0 mmol scale. The resulting mixture underwent purification via silica gel column chromatography using a hexane/ethyl acetate ratio of 10:1. This process yielded a white solid (112.4 mg, 0.48 mmol, 48% yield). ^1^H NMR (400 MHz, CDCl_3_) δ (ppm): 2.93 (t, *J* = 8.1 Hz, 4H), 1.85–1.78 (m, 4H), 1.47–1.39 (m, 4H), 1.36–1.21 (m, 8H), 0.88 (t, *J* = 6.8 Hz, 6H). ^13^C NMR (100 MHz, CDCl_3_) δ (ppm): 52.8, 31.3, 28.3, 22.4, 22.0, 14.0. HRMS (ESI): calcd for C_12_H_26_O_2_NaS [M + Na]^+^: 257.1547, found: 257.1545.

2-(Methylsulfonyl)-1*H*-benzimidazole (**3o**). This compound was synthesized following the general procedure using 2.5 equivalents of hydrogen chloride. The NMR yield was determined using 1,1,2,2-tetrachloroethane as an internal standard. ^1^H NMR (400 MHz, DMSO-*d*_6_) δ (ppm): 7.54–7.48 (m, 2H), 7.41–7.37 (m, 2H), 3.49 (s, 3H).

*N*-Acetylmethionine sulfone (**3p**). This compound was synthesized following the general procedure. The resulting mixture underwent purification using silica gel column chromatography (chloroform/methanol = 10:1, with 1% v/v acetic acid). This process yielded a yellow solid (78.2 mg, 0.35 mmol, 70% yield). ^1^H NMR (400 MHz, D_2_O) δ (ppm): 4.38–4.35 (m, 1H), 3.45–3.33 (m, 2H), 3.22 (s, 3H), 2.46–2.37 (m, 1H), 2.27–2.09 (m, 2H), 2.14 (s, 3H). ^13^C NMR (100 MHz, DMSO-*d*_6_) δ (ppm): 172.6, 169.7, 50.7, 50.6, 40.3, 24.0, 22.4. Mp: 198.1–199.0 °C, HRMS (MALDI): calcd for C_7_H_13_NO_5_NaS [M + Na]^+^: 246.0407, found: 246.0417.

## 4. Conclusions

We developed a versatile and efficient method for the selective oxidation of sulfides to sulfones by generating chlorine dioxide in situ from sodium chlorite and hydrochloric acid. This approach, conducted under mild conditions in organic solvents, provides high yields and excellent selectivity, demonstrated by the near-quantitative conversion of diphenyl sulfide to diphenyl sulfone. The method overcomes the limitations associated with aqueous oxidations, such as poor substrate solubility and undesired side reactions, while maintaining operational simplicity and scalability. Substrate scope evaluations suggest that the reaction is widely applicable, although substrates with strong electron-donating groups or significant steric hindrance tend to exhibit somewhat diminished reactivity. Overall, this oxidation protocol not only enhances the synthetic utility of organosulfur chemistry but also shows promise for applications in the syntheses of pharmaceuticals, natural products, and advanced materials. Future studies will focus on refining the reaction conditions and expanding the range of substrates.

## Figures and Tables

**Table 1:** Optimization of diphenyl sulfide oxygenation using NaClO_2_ in EtOAc.

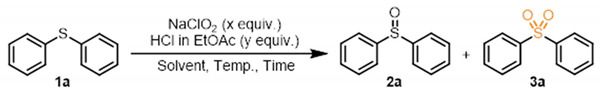									
Entry	x	y	Solvent	Conc. of 1a (M)	Temp. (°C)	Time	NMR Yield *^a^* (%)		
							1a	2a	3a
1	5.0	4.0	MeCN	0.040	25	1 h	0	0	96
2	5.0	4.0	EtOAc	0.040	25	1 h	0	0	96
3	5.0	4.0	Toluene	0.040	25	1 h	0	45	30
4 *^b^*	5.0	4.0	H_2_O	0.040	25	1 h	0	58	35
5	5.0	0	EtOAc	0.040	60	24 h	100	0	0
6	5.0	4.0	EtOAc	0.20	25	1 h	0	14	80
7	5.0	4.0	MeCN	0.20	25	1 h	0	0	95
8	5.0	4.0	MeCN	0.20	25	10 min	0	9	85
9	5.0	4.0	MeCN	0.20	25	1 h	0	43	50

*^a^* Determined using 1,1,2,2-tetrachloroethane as an internal standard. *^b^* HCl aq. instead of HCl in EtOAc.

## Data Availability

The Supplementary Materials are available free of charge on the website.

## Supplementary Materials

The following supporting information can be downloaded at https://www.mdpi.com/article/10.3390/molecules30091912/s1.
