# Supplementary material for: Sulfide Oxidation to Sulfone Using Sodium Chlorite and Hydrochloric Acid in Organic Solvents
Source: Molecules. 2025 Apr 25;30(9):1912. doi: 10.3390/molecules30091912 (PMC12073263; doi:10.3390/molecules30091912)

Supporting Information (SI)

Sulfide Oxidation to Sulfone Using Sodium Chlorite and Hydrochloric Acid in Organic Solvents

Yuki Itabashi 1, Shuto Ogata 2, Tsuyoshi Inoue 1,2, Haruyasu Asahara 1,2,* and Kei Ohkubo 1,*

1 Institute for Open and Transdisciplinary Research Initiatives (OTRI), The University of Osaka, 1-6 Yamada-oka, Suita 565-0871, Osaka, Japan

2 Graduate School of Pharmaceutical Sciences, The University of Osaka, 1-6 Yamada-oka, Suita 565-0871, Osaka, Japan

* Correspondence: asahara@phs.osaka-u.ac.jp (H.A.); ohkubo@irdd.osaka-u.ac.jp (K.O.)

Table of contents

1.	General Information	S3
2.	UV-Vis absorption spectral measurements	S3
3.	ESR Measurements	S3
4.	Synthetic procedures of bis(4-methoxyphenyl) sulfide	S3
5.	General procedure for the selective oxidation of sulfides	S4
6.	Optimization of solvents	S5
7.	Spectral data	S5
8.	NMR spectra	S10

1. General Information

Sodium chlorite (technical grade, 80%) was purchased from Sigma-Aldrich (St. Louis, MO, USA). HCl in ethyl acetate (1M) was purchased from TCI (Tokyo, Japan). Unless noted otherwise, all materials were purchased from Sigma-Aldrich (St. Louis, MO, USA), TCI (Tokyo, Japan), FUJIFILM Wako Pure Chemical (Osaka, Japan), and other commercial suppliers without further purification. Nuclear magnetic resonance (NMR) spectra were recorded using a Bruker AVANCE NEO 400 spectrometer (Billerica, MA, USA), with chemical shifts calibrated using residual undeuterated solvent ($CHCl_3$ at 7.26 ppm for ^1H NMR, 77.16 ppm for ^{13}C NMR; DMSO at 2.50 ppm for ^1H NMR, 39.52 ppm for ^{13}C NMR). The abbreviations for multiplicities are as follows: s (singlet), d (doublet), t (triplet), q (quartet), m (multiplet), and br (broad). High-resolution mass spectra were acquired with an AB SCEIX Triplet TOF 4600 mass spectrometer (Marsiling, Singapore).

2. UV-Vis Absorption Spectral Measurements

UV-Vis spectral measurements were conducted to determine the time taken to achieve the maximum generation of chlorine dioxide in the reaction between sodium chlorite and hydrogen chloride in acetonitrile. The spectra were captured using a JASCO V-750 UV-Vis Spectrophotometer. In a 9 mL screw-cap vial, sodium chlorite (57 mg, 0.50 mmol), hydrogen chloride in EtOAc solution (400 μL, 0.40 mmol), MeCN (600 μL) were combined. The mixture was then stirred for varying durations: 1 min, 10 min, 1 h, and 2 h at 25°C. Subsequently, the supernatant was diluted 20-fold, and UV-Vis spectroscopy was conducted at the same temperature.

3. ESR Measurements

$ClO_2^{\bullet}$ was produced by mixing $NaClO_2$ (0.14 mg) and HCl (1.0 mg) in EtOAc (5.0 mL) at 298 K. The resulting solution was then transferred to a quartz ESR capillary tube with an internal diameter of 1.8 mm. ESR spectra were recorded using a JEOL X-band spectrometer (JES-X310) under nonsaturating microwave power conditions. The modulation magnitude was selected to enhance resolution and signal-to-noise ratio (S/N) without altering the maximum slope linewidth (ΔH_{msl}) of the ESR signals. The *g* values and hyperfine coupling (*hfc*) constants were calibrated using a Mn^{2+} marker and determined through computer simulation software (ver. 2.4.4) provided by JEOL Ltd. (Tokyo, Japan).

4. Synthetic Procedures of Bis(4-methoxyphenyl) Sulfide

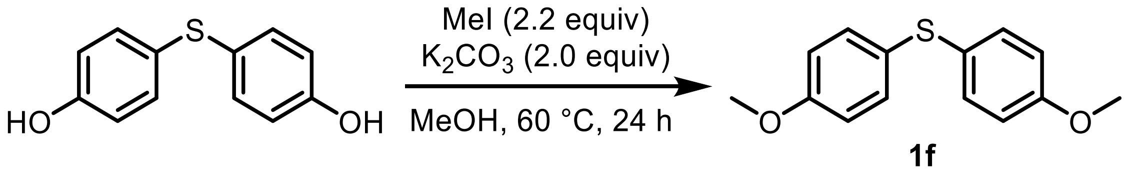

1f

To a 30 mL test tube, bis(4-hydroxyphenyl) sulfide (1.31 g, 6.0 mmol), potassium carbonate (1.70 g, 12 mmol), MeOH (12 mL), and iodomethane (1.85 g, 13 mmol) were added. The mixture was stirred for 24 h at 60 °C. The reaction was quenched with water (28 mL) and a solution of aqueous 36% hydrogen chloride (430 μL). The solution was extracted with $CHCl_3$ (28 mL × 2), and the combined organic layers were dried over anhydrous Na_2SO_4. The solvent was removed under reduced pressure. Subsequently, the mixture underwent purification by silica gel column chromatography using hexane/EtOAc (5:1) as the eluent, resulting in the isolation of the desired product **1f** in the form of a white solid (1.12 g, 4.5 mmol, 76% yield). **^{1}H NMR (400 MHz, $CDCl_3$)** δ(ppm): 7.28 (d, J = 8.8 Hz, 4H), 6.84 (d, J = 8.8 Hz, 4H), 3.79 (s, 6H). **^{13}C NMR (100 MHz, $CDCl_3$)** δ(ppm): 159.0, 132.8, 127.5, 114.8, 55.4. **HRMS (MALDI)**: calcd for $C_{14}H_{15}O_2S$ $[M+H]^+$: 246.0709, found: 246.0711.

5. General Procedure for the Selective Oxidation of Sulfides

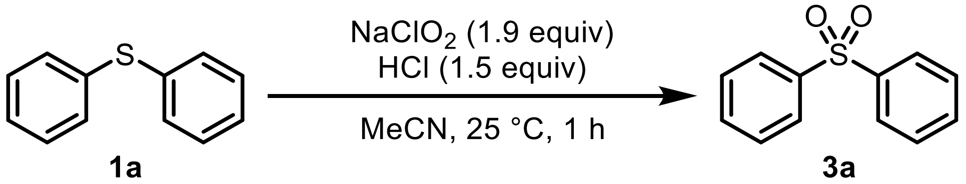

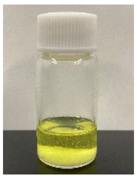

To a 6 mL screw vial, diphenyl sulfide **1a** (93.1 mg, 0.50 mmol), sodium chlorite (105 mg, 0.95 mmol), MeCN (1.8 mL), and a solution of hydrogen chloride in ethyl acetate (750 μL, 0.75 mmol) were added. The mixture was stirred for 1 h at 25 °C. Subsequently, the mixture underwent purification by silica gel column chromatography using EtOAc as the eluent. After removal of the solvent under reduced pressure using a rotary evaporator, the desired product **3a** was obtained as a white solid (104 mg, 0.48 mmol, 95% yield).

Procedure for the Synthesis of 3a on a Gram-scale: To a 30 mL screw vial, diphenyl sulfide **1a** (1.00 g, 5.4 mmol), sodium chlorite (1.13 g, 10 mmol), MeCN (19 mL) and a solution of hydrogen chloride in ethyl acetate (8.05 mL, 8.1 mmol) were added. The mixture was stirred for 1 h at 25 °C. Subsequently, the mixture underwent purification by silica gel column chromatography using EtOAc as the eluent, resulting in the isolation of the desired product **3a** in the form of a white solid (1.12 g, 5.0 mmol, 94% yield).

6. Optimization of solvents

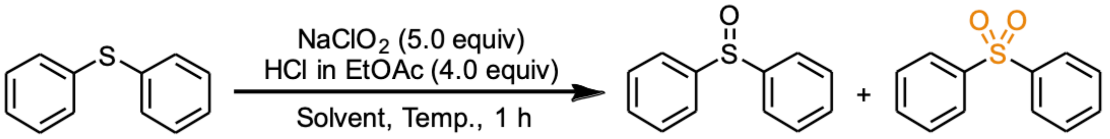

1a (0.040 M) **2a** **3a**

Entry	Solvent	Temp. (°C)	NMR Yield (%)		
			**1a**	**2a**	**3a**
1	MeCN	25	0	0	96
2	EtOAc	25	0	0	96
3	CH_2Cl_2	25	0	0	95
4	Toluene	25	0	45	30
5	Toluene	60	0	0	90

High yields of **3a** were obtained when MeCN, EtOAc, or CH_2Cl_2 was used as the solvent. In contrast, switching the solvent to toluene resulted in a significantly reduced yield of **3a** (30%) along with the formation of **2a** in 45% yield. Notably, performing the reaction at 60 °C led to an improved yield of **3a** (90%) without any detectable formation of **2a**.

7. Spectral Data

Diphenyl sulfone (**3a**)

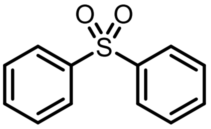

This compound was synthesized following the general procedure. A white solid was obtained (104 mg, 0.48 mmol, 95% yield). **^{1}H NMR (400 MHz, $CDCl_3$)** δ (ppm): 8.04–7.88 (m, 4H), 7.65–7.43 (m, 6H). **^{13}C NMR (100 MHz, $CDCl_3$)** δ (ppm): 144.4, 132.8, 126.2, 126.0. **HRMS(MALDI)**: calcd for $C_{12}H_{11}O_2S$ $[M+H]^+$: 219.0474, found: 219.0476.

Bis(4-chlorophenyl) sulfone (**3b**)

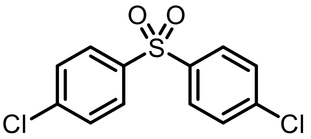

This compound was synthesized following the general procedure. The resulting mixture underwent purification via silica gel column chromatography using a hexane/ethyl acetate ratio of 4:1. This process yielded a white solid (123 mg, 0.43 mmol, 86% yield). **^{1}H NMR (400 MHz, $CDCl_3$)** δ (ppm): 7.86 (d, J = 8.8 Hz, 4H), 7.48 (d, J = 8.8 Hz, 4H). **^{13}C NMR (100 MHz, $CDCl_3$)** δ (ppm): 140.3, 139.8, 129.8, 129.2. **HRMS (ESI)**: calcd for $C_{12}H_8O_2NaSCl_2$ $[M+Na]^+$:308.9517, found: 308.9514.

Bis(4-bromophenyl) sulfone (**3c**)

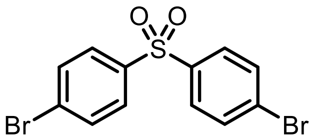

This compound was synthesized following the general procedure. The resulting mixture underwent purification via silica gel column chromatography using a hexane/ethyl acetate ratio of 4:1. This process yielded a white solid (153 mg, 0.41 mmol, 82% yield). **^{1}H NMR (400 MHz, CDCl$_3$)** δ (ppm): 7.78 (d, *J* = 8.8 Hz, 4H), 7.65 (d, *J* = 8.8 Hz, 4H). **^{13}C NMR (100 MHz, CDCl$_3$)** δ (ppm): 140.3, 132.8, 129.3, 128.9. **HRMS (ESI)**: calcd for $C_{12}H_8O_2NaSBr_2$ [M+Na]$^+$: 396.8504, found: 396.8504.

Bis(4-nitrophenyl) sulfone (**3d**)

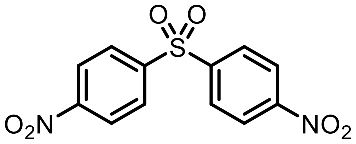

This compound was synthesized following the general procedure using 3.8 equivalents of $NaClO_2$ and 3.0 equivalents of hydrogen chloride. A yellow solid was obtained (111 mg, 0.36 mmol, 72% yield). **^{1}H NMR (400 MHz, DMSO-*d$_6$*)** δ (ppm): 8.42 (d, *J* = 9.0 Hz, 4H), 8.30 (d, *J* = 9.0 Hz, 4H). **^{13}C NMR (100 MHz, DMSO-*d$_6$*)** δ (ppm): 150.8, 144.8, 129.6 125.2. **HRMS (ESI)**: calcd for $C_{12}H_8N_2O_6NaS$ [M+Na]$^+$: 331.0001, found: 331.0003.

Bis(4-methoxyphenyl) sulfone (**3f**)

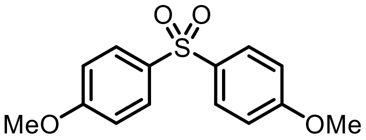

This compound was synthesized following the general procedure. The resulting mixture underwent purification via silica gel column chromatography using a hexane/ethyl acetate ratio of 4:1. This process yielded an orange solid (83.4 mg, 0.30 mmol, 60% yield). **^{1}H NMR (400 MHz, CDCl$_3$)** δ (ppm): 7.83 (d, *J* = 9.0 Hz, 4H), 6.93 (d, *J* = 9.0 Hz, 4H), 3.81 (s, 6H). **^{13}C NMR (100 MHz, CDCl$_3$)** δ (ppm): 163.1, 133.9, 129.5, 114.5, 55.7. **HRMS (MALDI)**: calcd for $C_{14}H_{15}O_4S$ [M+H]$^+$: 279.0686, found: 279.0684.

Methyl phenyl sulfone (**3g**)

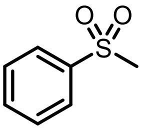

This compound was synthesized following the general procedure. The resulting mixture underwent purification via silica gel column chromatography using a hexane/ethyl acetate ratio of 4:1. This process yielded an orange solid (31.3 mg, 0.20 mmol, 40% yield). **^{1}H NMR (400 MHz, CDCl$_3$)** δ (ppm): 7.99–7.87 (m, 2H), 7.71–7.61 (m, 1H), 7.60–7.50 (m, 2H), 3.04 (s, 3H). **^{13}C NMR (100 MHz, CDCl$_3$)** δ (ppm): 140.5, 133.7, 129.4, 127.3, 44.5. **HRMS (MALDI)**: calcd for $C_7H_8O_2NaS$ [M+Na]$^+$: 179.0137, found: 179.0136.

Methyl 4-tolyl sulfone (**3h**)

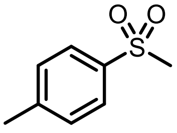

This compound was synthesized following the general procedure. The resulting mixture underwent purification via silica gel column chromatography using a hexane/ethyl acetate ratio of 4:1. This process yielded a white solid (32.3 mg, 0.19 mmol, 38% yield). **^{1}H NMR (400 MHz, CDCl$_3$)** δ (ppm): 7.81 (d, *J* = 8.0 Hz, 2H), 7.35 (d, *J* = 8.0 Hz, 2H), 3.02 (s, 3H), 2.44 (s, 3H). **^{13}C NMR (100 MHz, CDCl$_3$)** δ (ppm): 144.7, 137.7, 130.0, 127.4, 44.7, 21.7. **HRMS (MALDI)**: calcd for $C_8H_{10}O_2NaS$ [M+Na]$^+$: 193.0294, found: 193.0296.

Dibenzyl sulfone (**3i**)

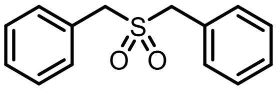

This compound was synthesized following the general procedure on a 2.0 mmol scale. The resulting mixture underwent purification via silica gel column chromatography using a hexane/ethyl acetate ratio of 7:1. This process yielded a yellow solid (34.2 mg, 0.14 mmol, 7% yield). **^{1}H NMR (400 MHz, CDCl$_3$)** δ (ppm): 7.51–7.29 (m, 10H), 4.13 (s, 4H). **^{13}C NMR (100 MHz, CDCl$_3$)** δ (ppm): 130.9, 129.1, 129.1, 127.6, 58.0. **HRMS (ESI)**: calcd for $C_{14}H_{14}O_2NaS$ [M+Na]$^+$: 269.0608, found: 269.0606.

Benzyl methyl sulfone (**3j**)

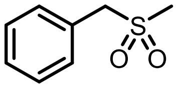

This compound was synthesized following the general procedure. The NMR yield was determined using 1,1,2,2-tetrachloroethane as an internal standard. **^{1}H NMR (400 MHz, CDCl$_3$)** δ (ppm): 7.45–7.35 (m, 5H), 4.24 (s, 2H), 3.30 (s, 3H). **^{13}C NMR (100 MHz, CDCl$_3$)** δ (ppm): 130.6, 129.2, 128.3, 128.2, 61.3, 39.1. **HRMS(MALDI)**: $C_8H_{10}O_2NaS$ [M+Na]$^+$: 193.0294, found: 193.0290.

Dibenzothiophene-5,5-dioxide (**3k**)

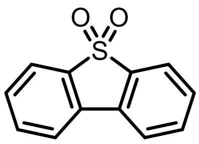

This compound was synthesized following the general procedure using 3.8 equivalents of $NaClO_2$ and 3.0 equivalents of hydrogen chloride. A white solid was obtained (97.2 mg, 0.45 mmol, 90% yield). **^{1}H NMR (400 MHz, CDCl$_3$)** δ (ppm): 7.88–7.72 (m, 4H), 7.71–7.56 (m, 2H), 7.55–7.45 (m, 2H). **^{13}C NMR (100 MHz, CDCl$_3$)** δ (ppm): 137.7, 134.0, 131.6, 130.4, 122.2, 121.7. **HRMS (MALDI)**: calcd for $C_{12}H_9O_2S$ [M+H]$^+$: 217.0318, found: 217.0317.

Benzo[*b*]naphtho[1,2-*d*]thiophene-11,11-dioxide (**3l**)

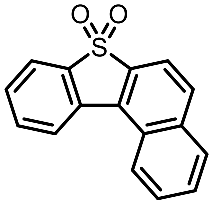

This compound was synthesized following the general procedure using 3.8 equivalents of $NaClO_2$ and 3.0 equivalents of hydrogen chloride. The NMR yield was determined using 1,1,2,2-tetrachloroethane as an internal standard. **^{1}H NMR (400 MHz, $CDCl_3$)** δ (ppm): 8.79–8.75 (m, 1H), 8.50–8.47 (m, 1H), 8.44 (d, *J* = 7.6 Hz, 1H), 8.06–7.98 (m, 2H), 7.92 (d, *J* = 7.6 Hz, 1H), 7.86–7.79 (m, 2H), 7.74 (ddd, *J* = 1.2, 7.6, 7.6 Hz, 1H), 7.60 (ddd, *J* = 0.7, 7.6, 7.6 Hz, 1H). **HRMS (ESI)**: calcd for $C_{16}H_{10}O_2NaS$ $[M+Na]^+$: 289.0279, found: 289.0293.

tert-Butyl methyl sulfone (**3m**)

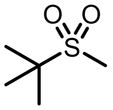

This compound was synthesized following the general procedure. The NMR yield was determined using 1,1,2,2-tetrachloroethane as an internal standard. **^{1}H NMR (400 MHz, $CDCl_3$)** δ (ppm): 2.81 (s, 3H), 1.42 (s, 9H). **HRMS (ESI)**: calcd for $C_5H_{12}O_2NaS$ $[M+Na]^+$: 159.0455, found: 159.0458.

Dihexyl sulfone (**3n**)

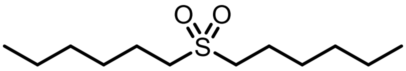

This compound was synthesized following the general procedure on a 1.0 mmol scale. The resulting mixture underwent purification via silica gel column chromatography using a hexane/ethyl acetate ratio of 10:1. This process yielded a white solid (112.4 mg, 0.48 mmol, 48% yield). **^{1}H NMR (400 MHz, $CDCl_3$)** δ (ppm): 2.93 (t, *J* = 8.1 Hz, 4H), 1.85–1.78 (m, 4H), 1.47–1.39 (m, 4H), 1.36–1.21 (m, 8H), 0.88 (t, *J* = 6.8 Hz, 6H). **^{13}C NMR (100 MHz, $CDCl_3$)** δ (ppm): 52.8, 31.3, 28.3, 22.4, 22.0, 14.0. **HRMS (ESI)**: calcd for $C_{12}H_{26}O_2NaS$ $[M+Na]^+$: 257.1547, found: 257.1545.

2-(Methylsulfonyl)-1*H*-benzimidazole (**3o**)

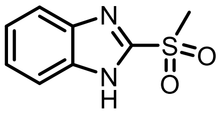

This compound was synthesized following the general procedure using 2.5 equivalents of hydrogen chloride. The NMR yield was determined using 1,1,2,2-tetrachloroethane as an internal standard. **^{1}H NMR (400 MHz, DMSO-d_6)** δ (ppm): 7.54–7.48 (m, 2H), 7.41–7.37 (m, 2H), 3.49 (s, 3H).

N-Acetylmethionine sulfone (**3p**)

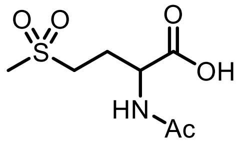

This compound was synthesized following the general procedure. The resulting mixture underwent purification using silica gel column chromatography (chloroform/methanol = 10:1, with 1% v/v acetic acid). This process yielded a yellow solid (78.2 mg, 0.35 mmol, 70% yield). **^{1}H NMR (400 MHz, D_2O)** δ (ppm): 4.38–4.35 (m, 1H), 3.45–3.33 (m, 2H), 3.22 (s, 3H), 2.46–2.37 (m, 1H), 2.27–2.09 (m, 2H), 2.14 (s, 3H). **^{13}C NMR (100 MHz, DMSO-*d*$_6$)** δ (ppm): 172.6, 169.7, 50.7, 50.6, 40.3, 24.0, 22.4. Mp: 198.1–199.0 °C, **HRMS (MALDI)**: calcd for $C_7H_{13}NO_5NaS$ $[M+Na]^+$: 246.0407, found: 246.0417.

7. NMR Spectral Data

^{1}H NMR (400 MHz, $CDCl_3$, 25 °C) of **1f**

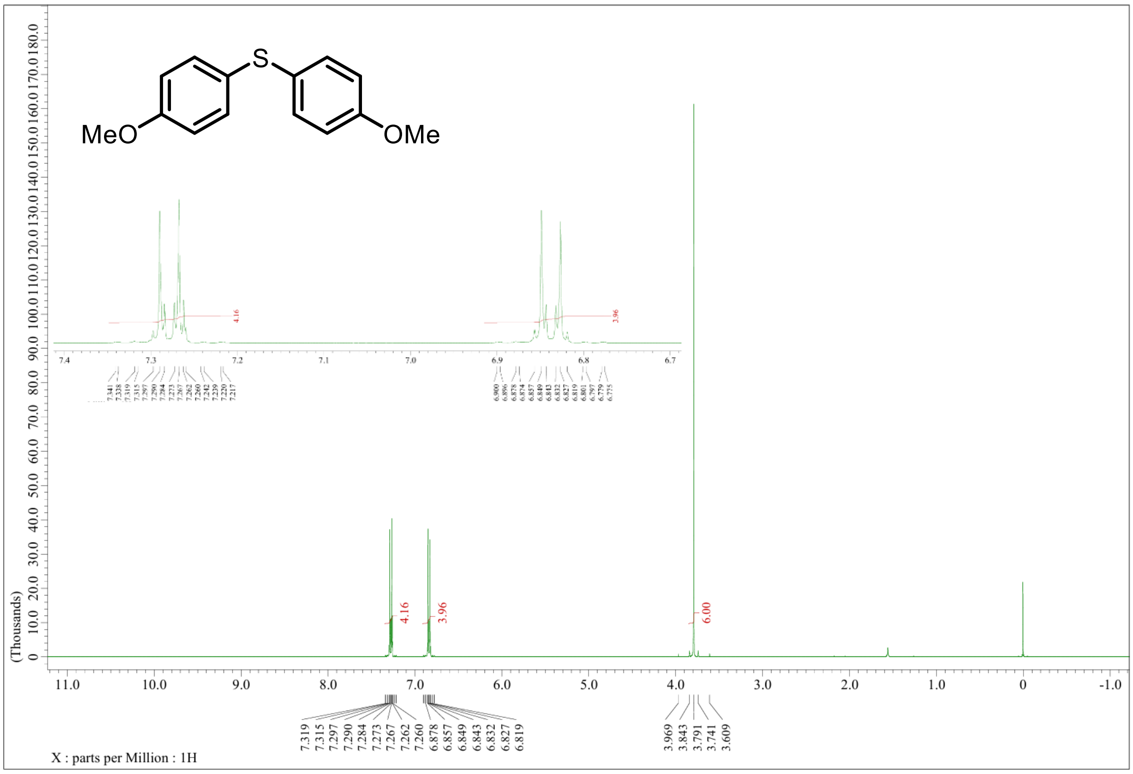

^{13}C NMR (100 MHz, $CDCl_3$, 25 °C) of **1f**

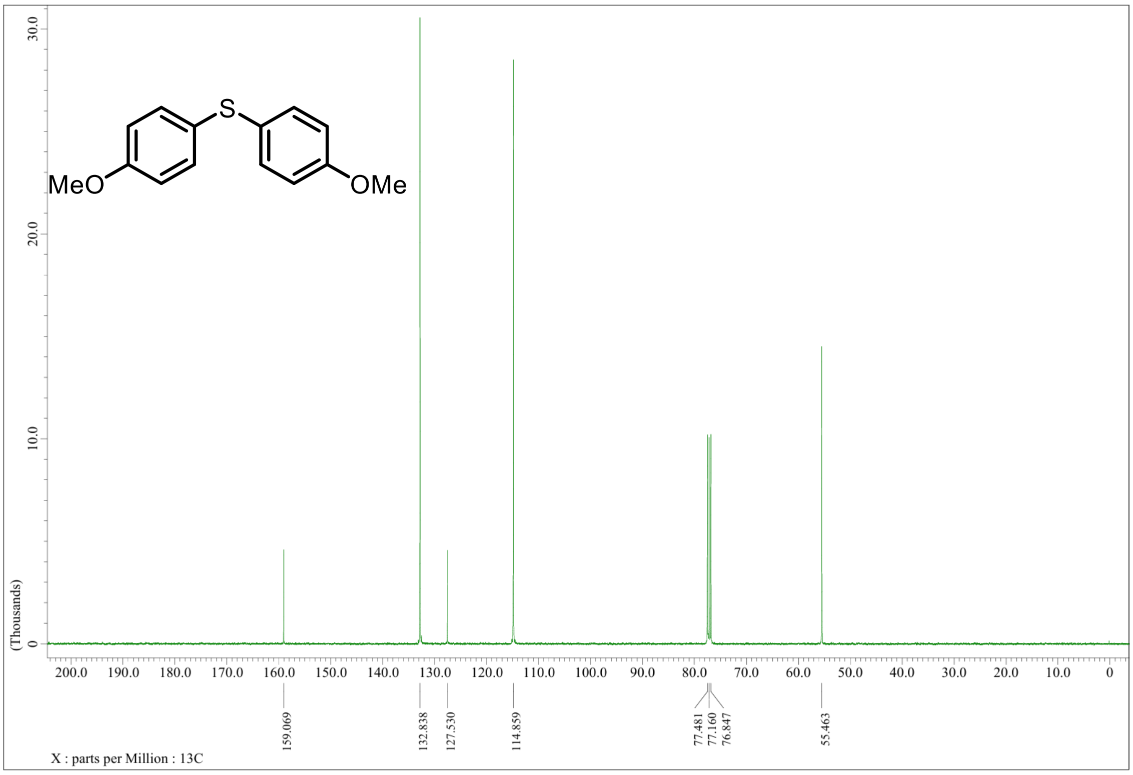

^{1}H NMR (400 MHz, $CDCl_3$, 25 °C) of **3a**

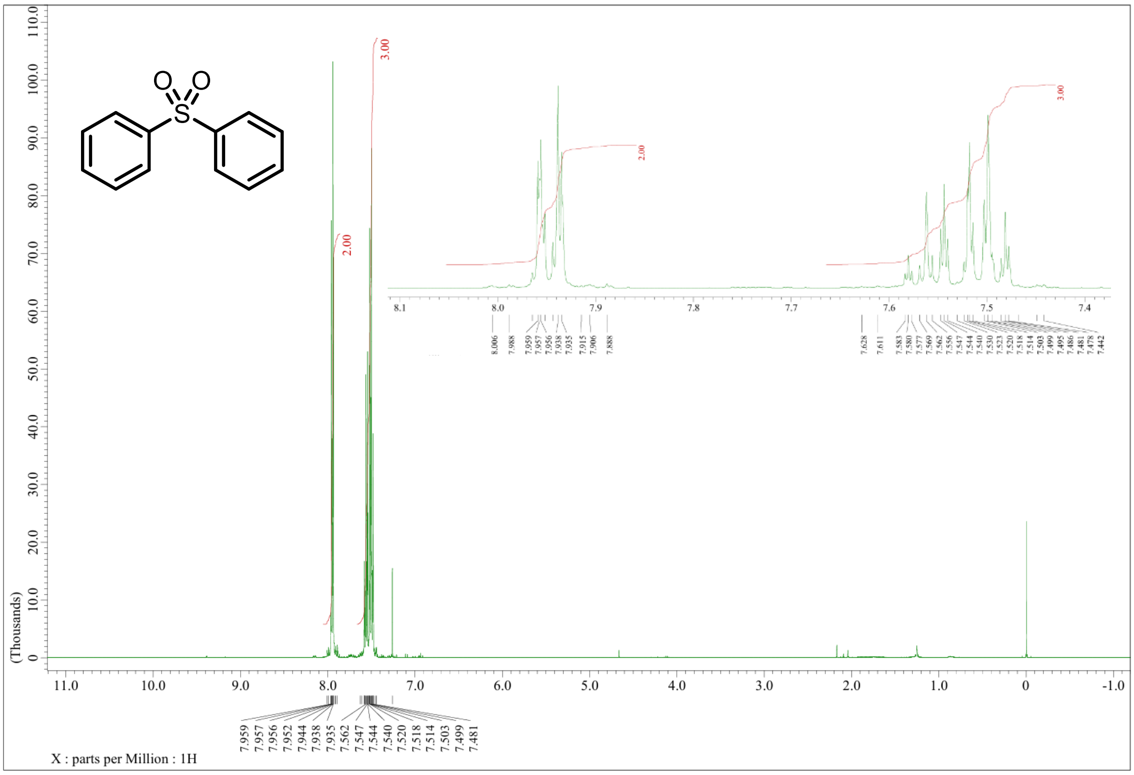

^{13}C NMR (100 MHz, $CDCl_3$, 25 °C) of **3a**

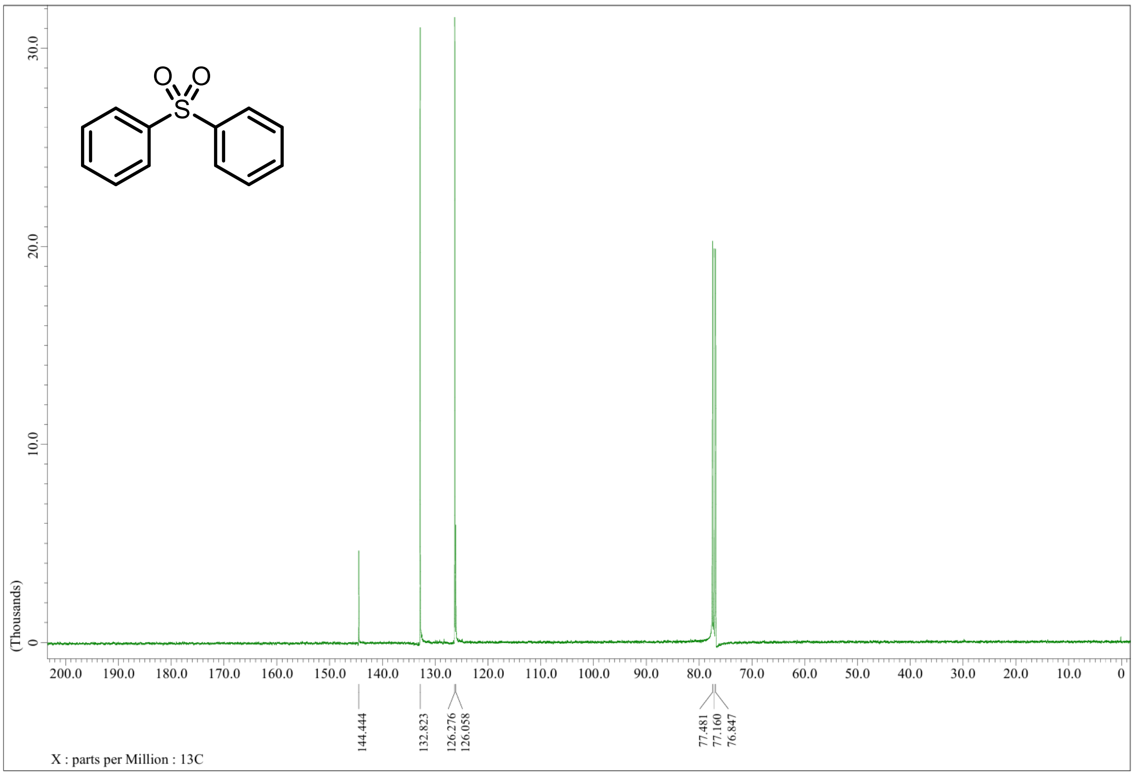

^{1}H NMR (400 MHz, $CDCl_3$, 25 °C) of **3b**

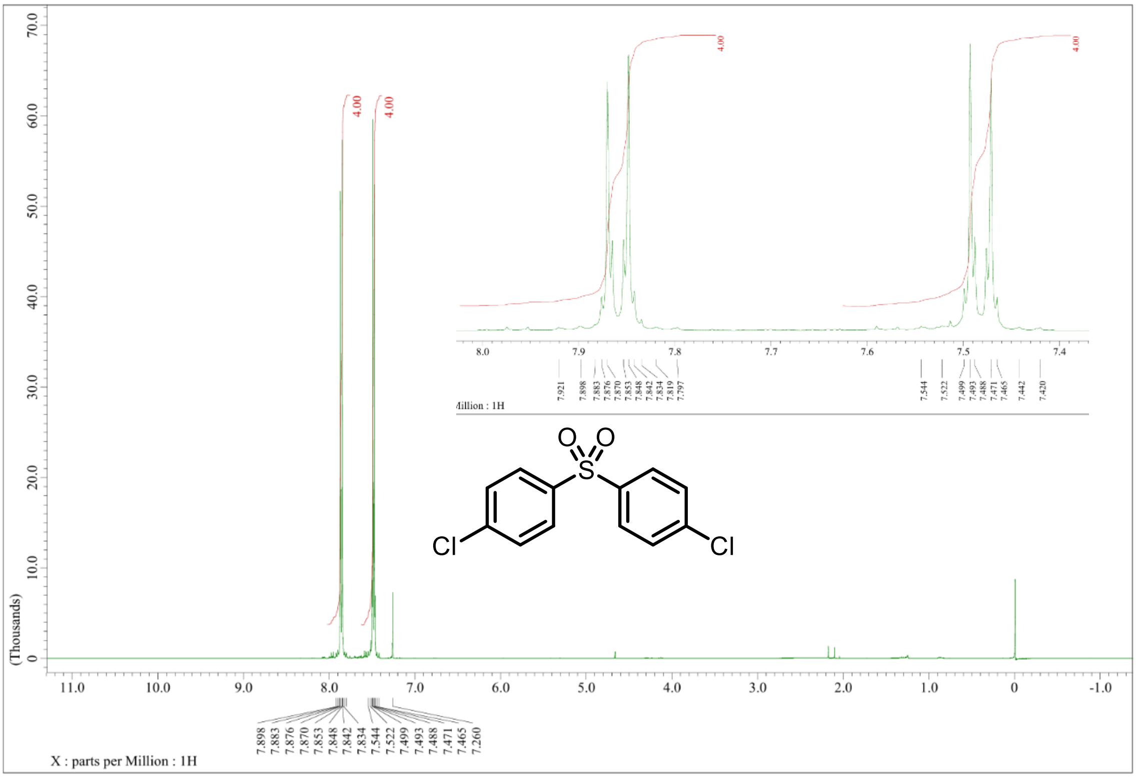

^{13}C NMR (100 MHz, $CDCl_3$, 25 °C) of **3b**

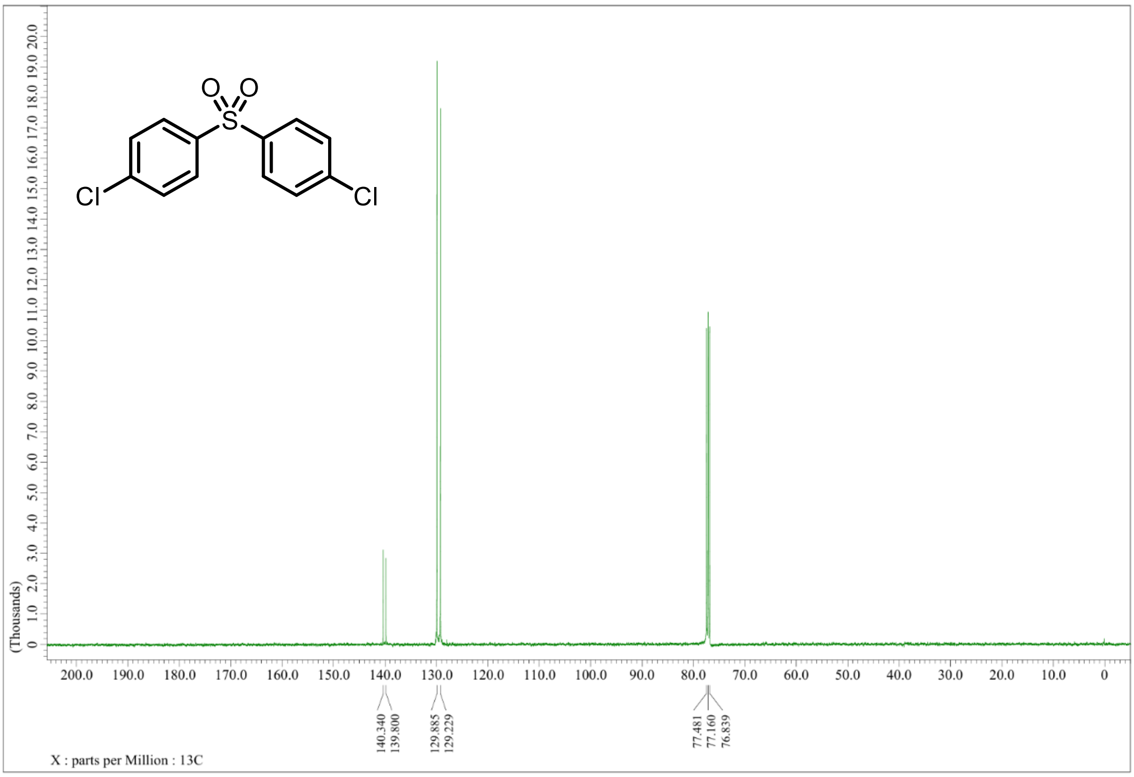

^{1}H NMR (400 MHz, $CDCl_3$, 25 °C) of **3c**

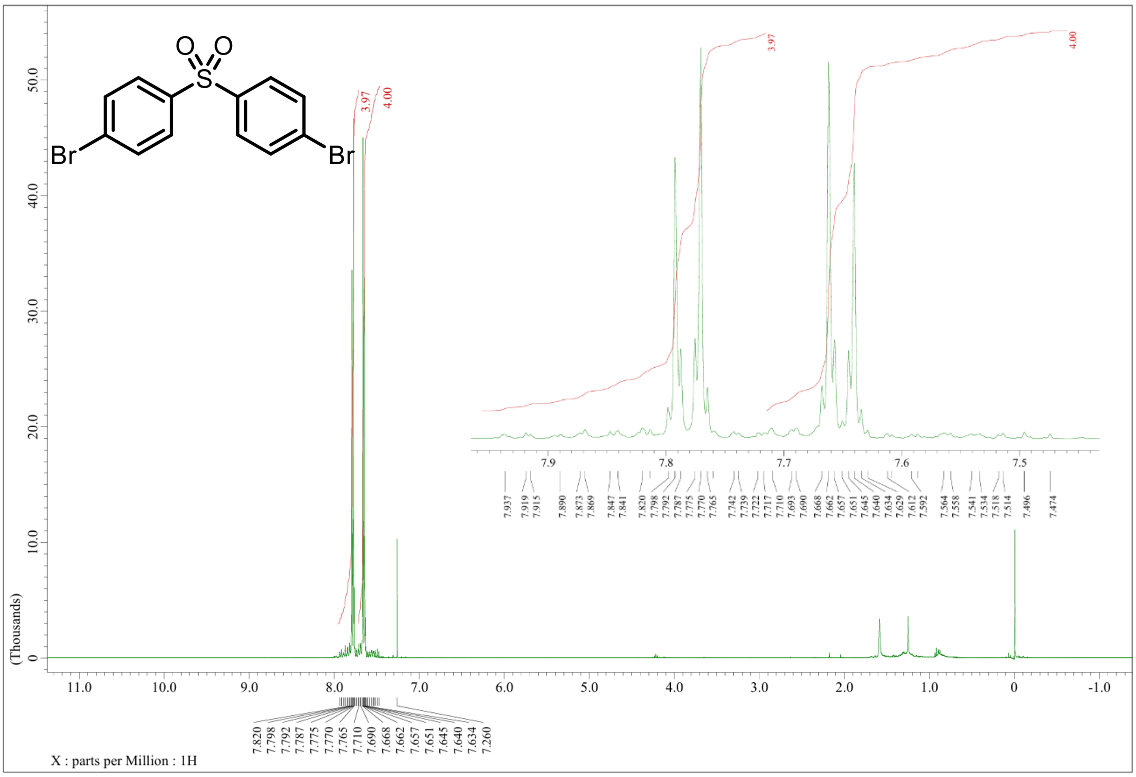

^{13}C NMR (100 MHz, $CDCl_3$, 25 °C) of **3c**

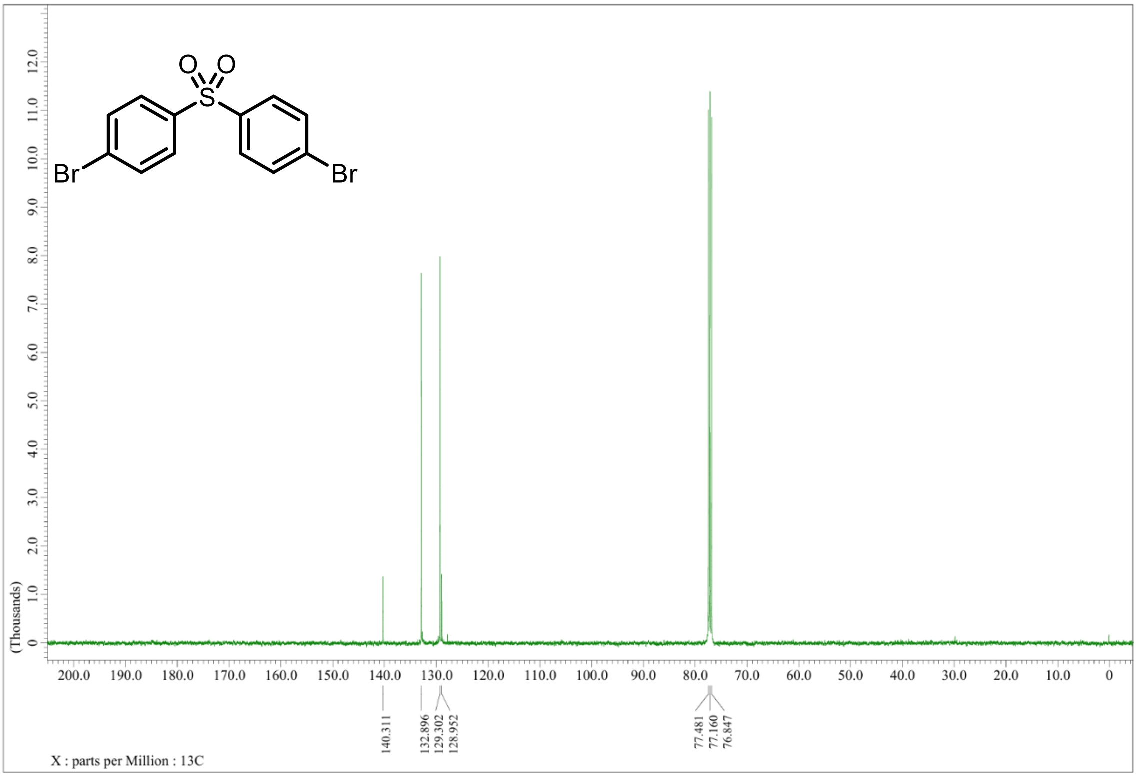

^{1}H NMR (400 MHz, DMSO-d_6, 25 °C) of **3d**

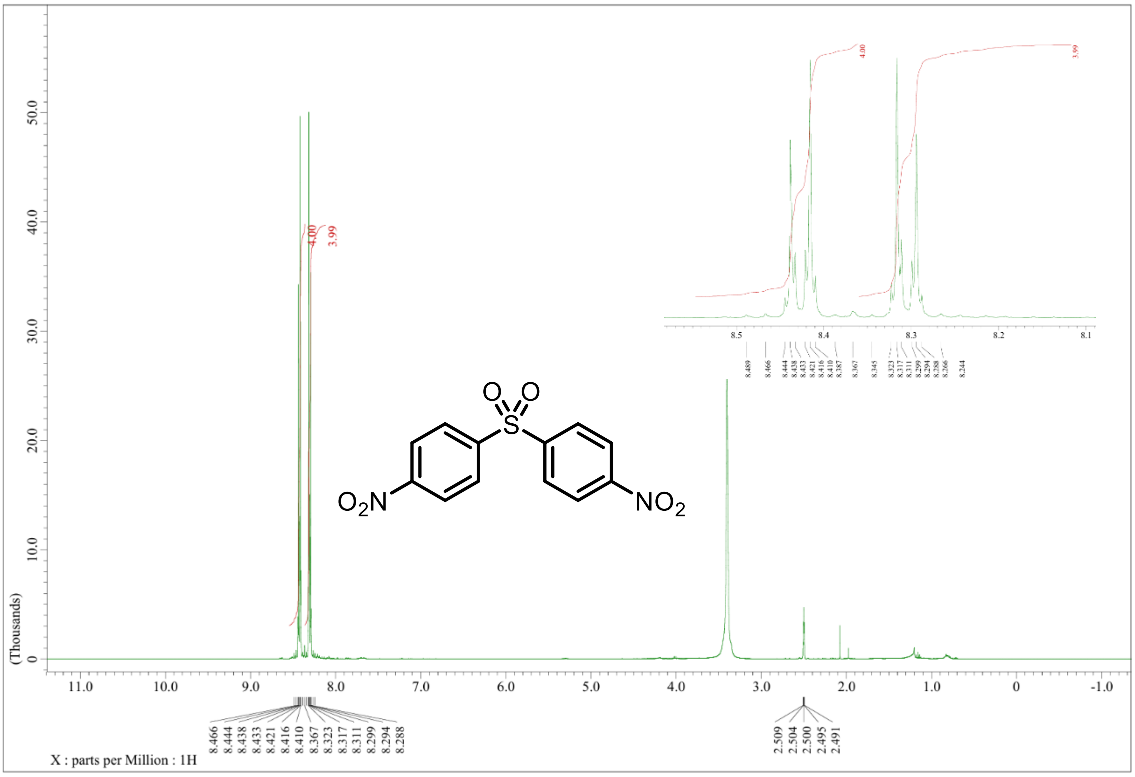

^{13}C NMR (100 MHz, DMSO-d_6, 25 °C) of **3d**

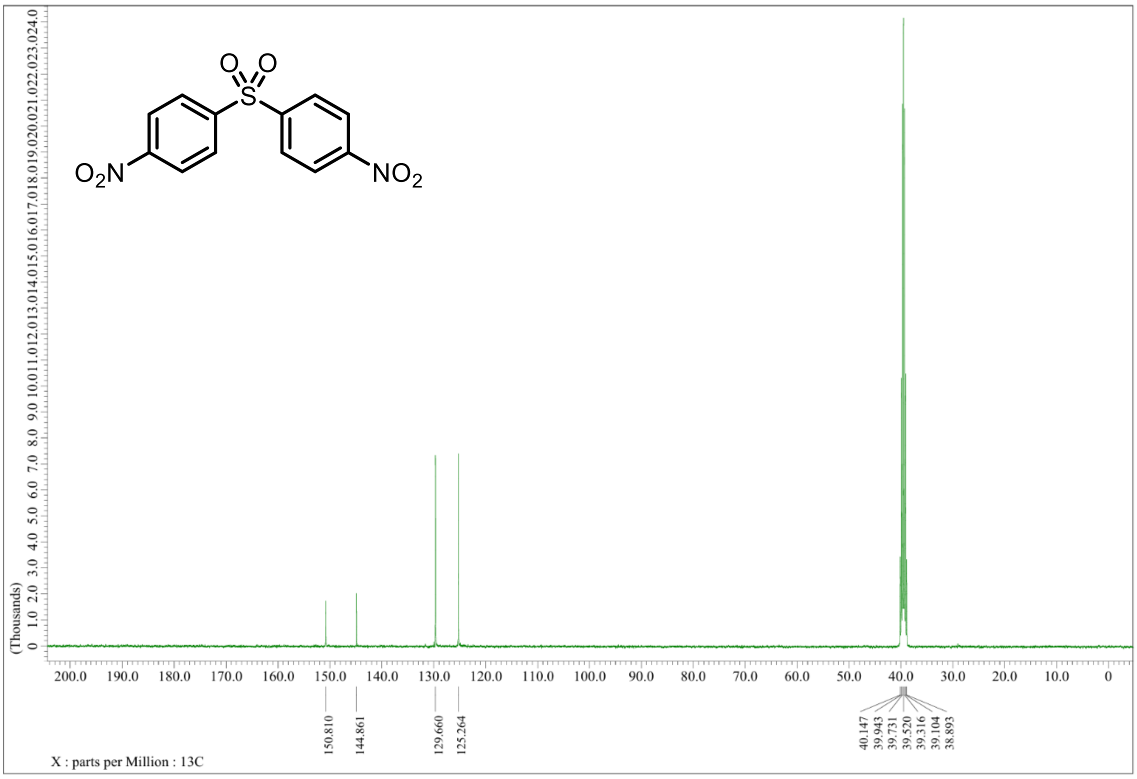

^{1}H NMR (400 MHz, $CDCl_3$, 25 °C) of **3f**

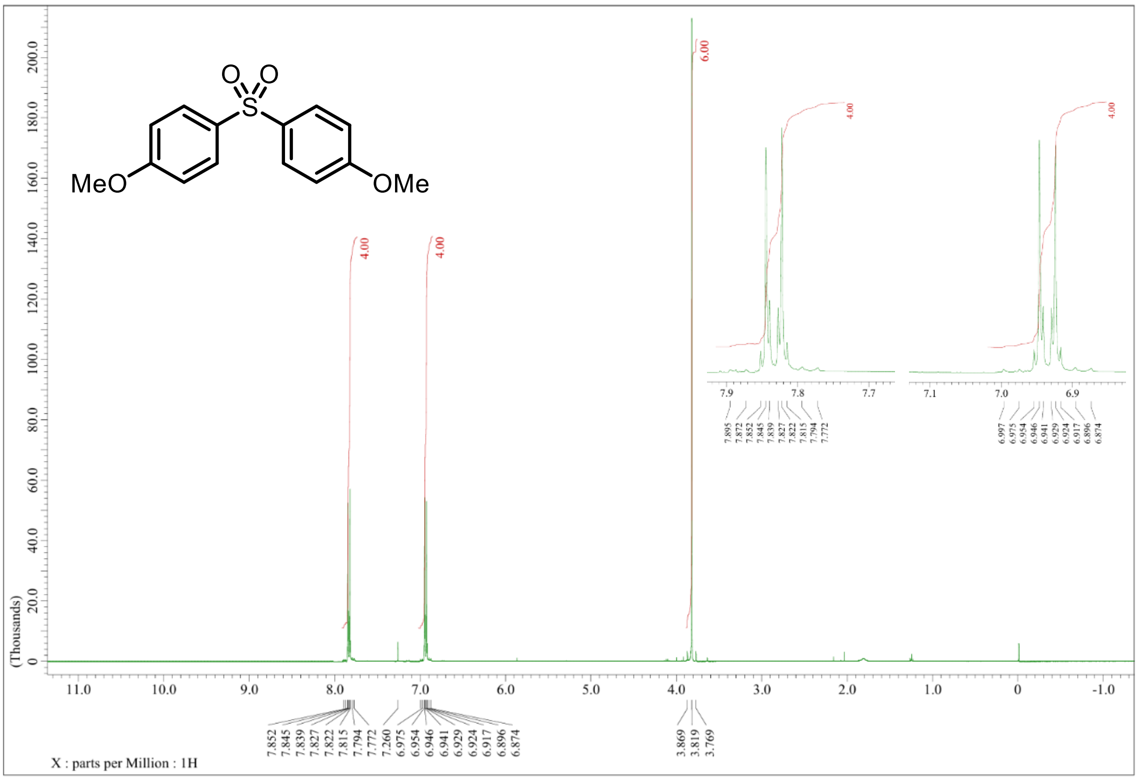

^{13}C NMR (100 MHz, $CDCl_3$, 25 °C) of **3f**

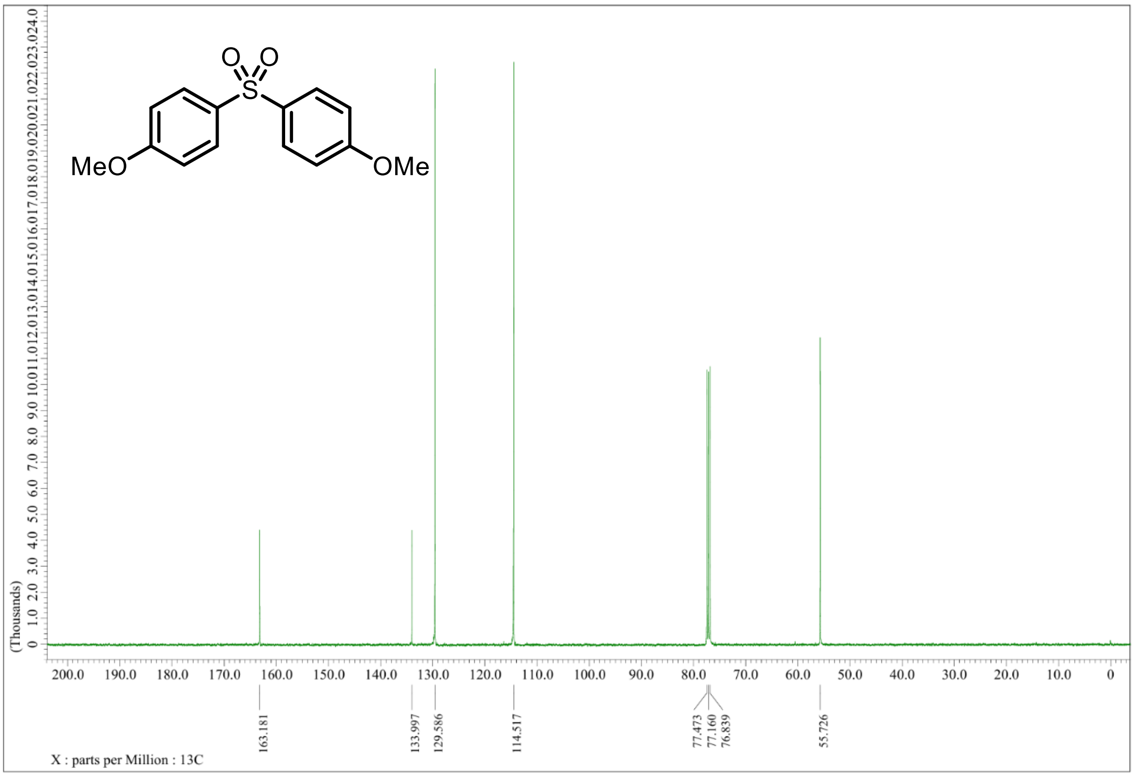

^{1}H NMR (400 MHz, $CDCl_3$, 25 °C) of **3g**

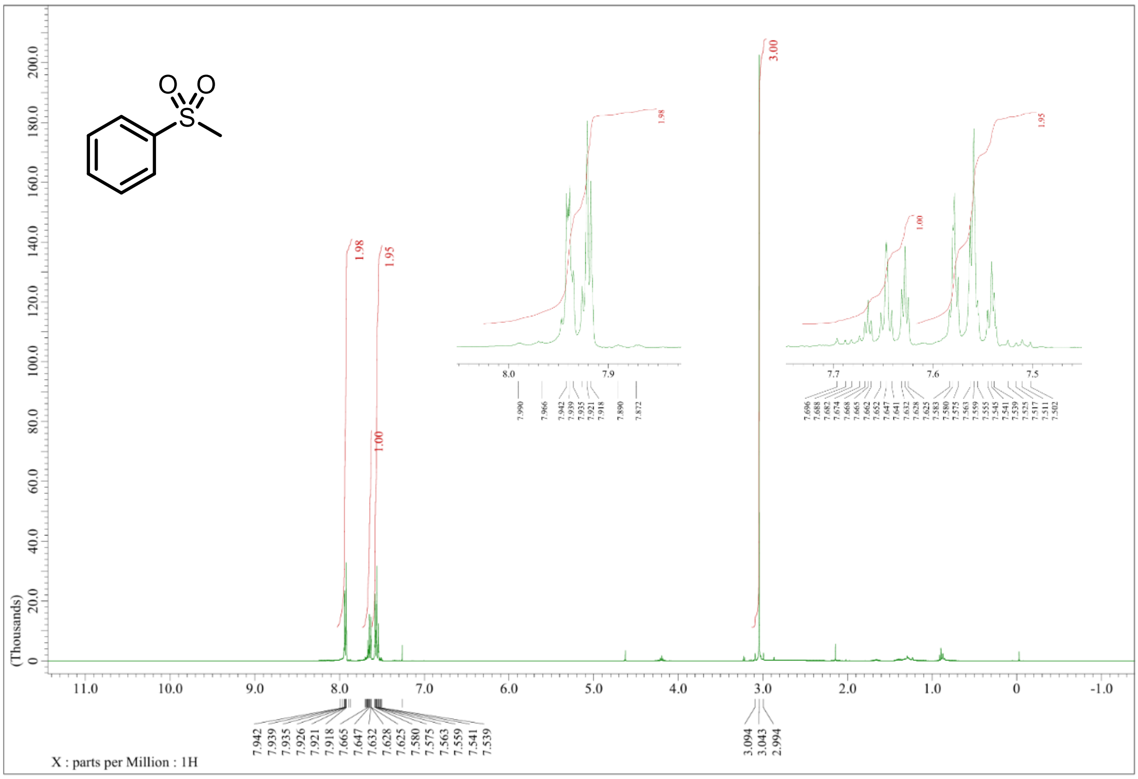

^{13}C NMR (100 MHz, $CDCl_3$, 25 °C) of **3g**

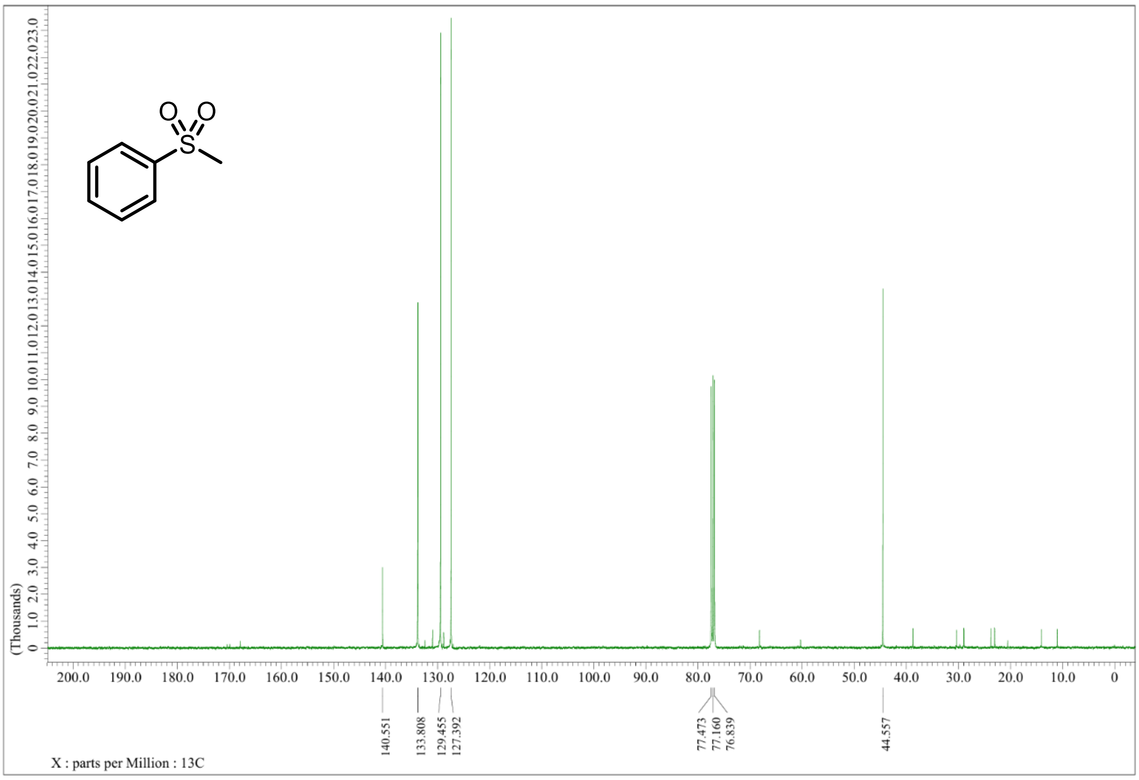

^{1}H NMR (400 MHz, $CDCl_3$, 25 °C) of **2h**

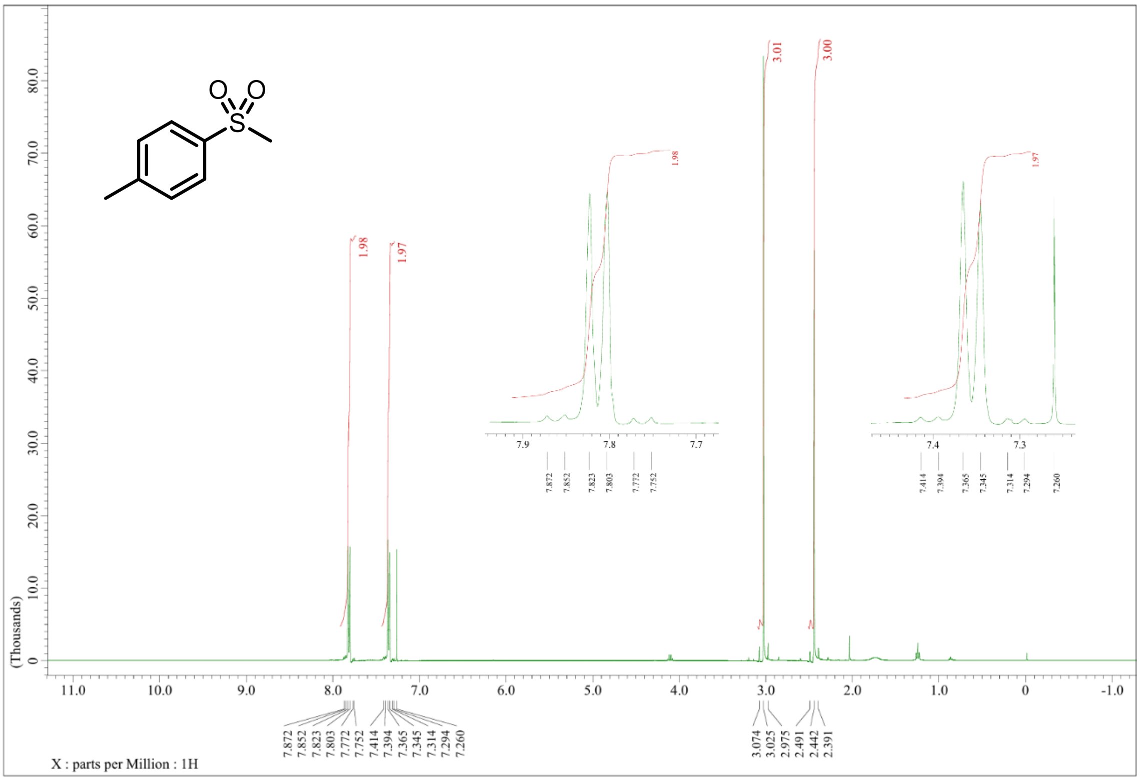

^{13}C NMR (100 MHz, $CDCl_3$, 25 °C) of **2h**

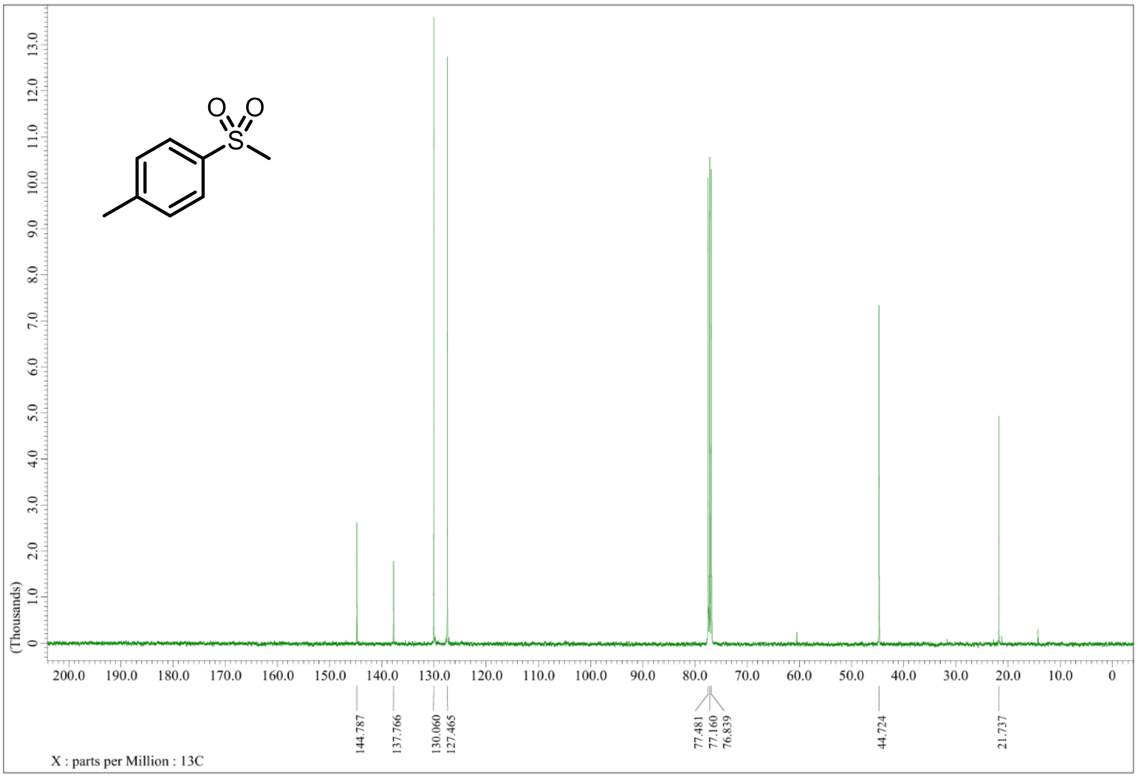

^{1}H NMR (400 MHz, $CDCl_3$, 25 °C) of **3i**

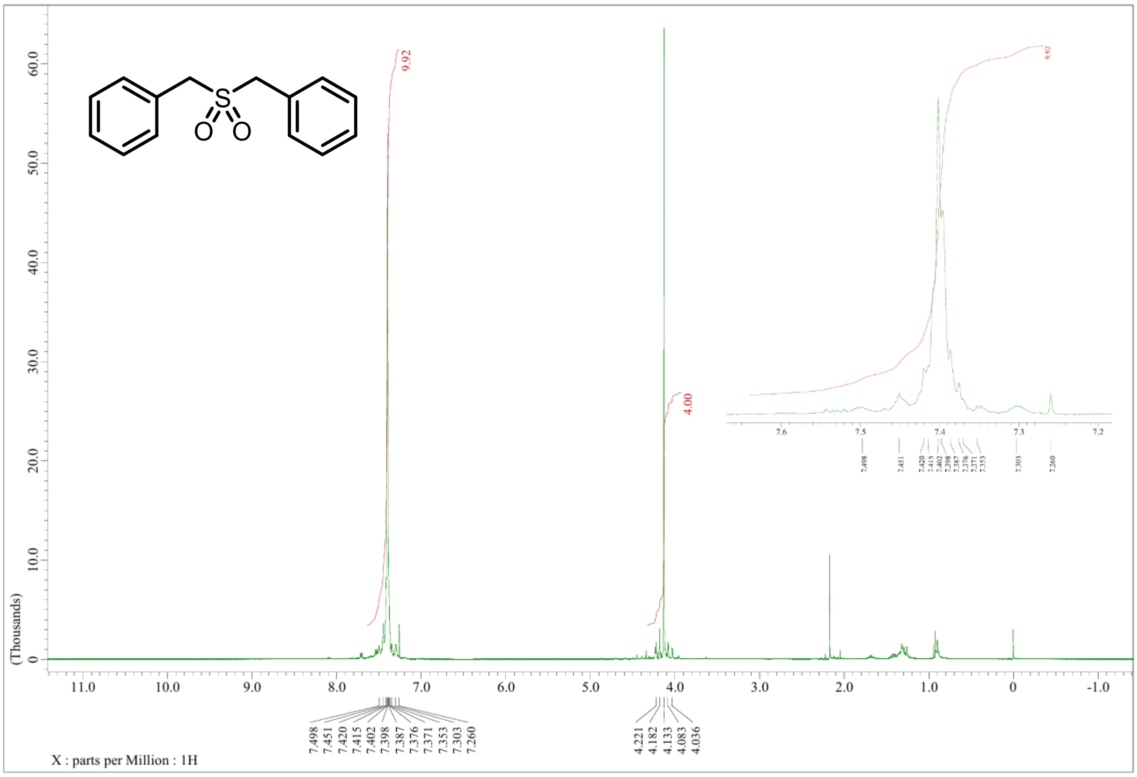

^{13}C NMR (100 MHz, $CDCl_3$, 25 °C) of **3n**

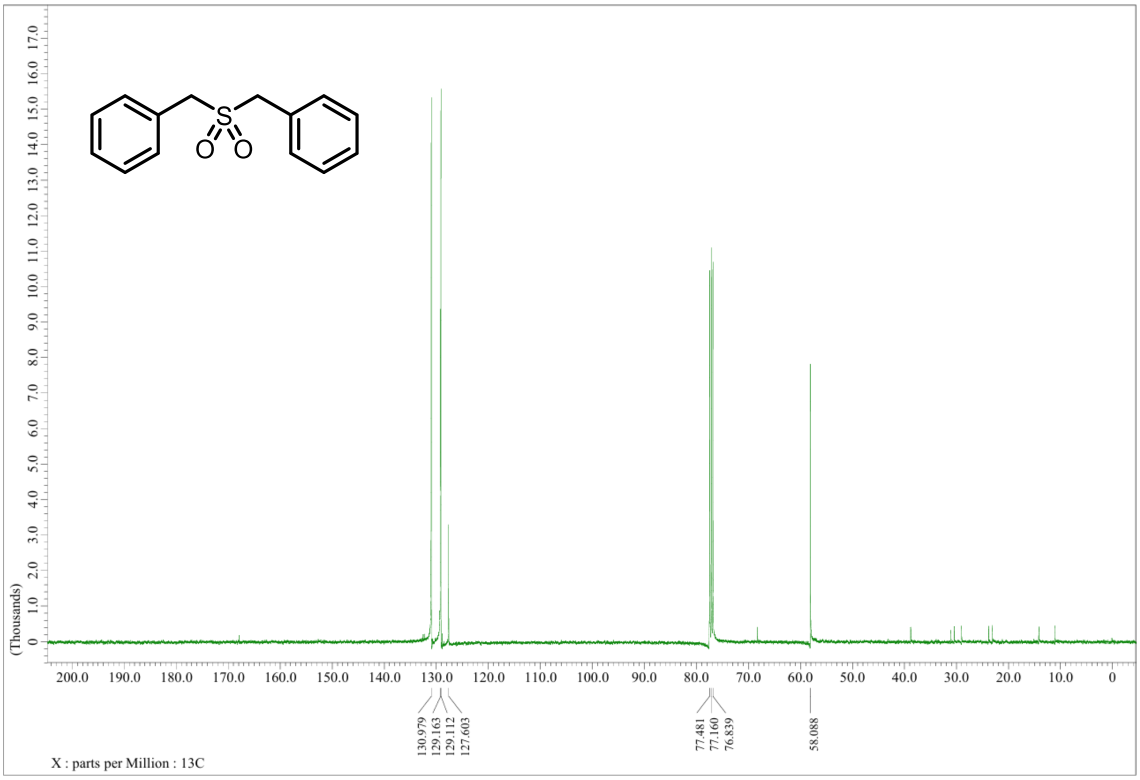

^{1}H NMR (400 MHz, $CDCl_3$, 25 °C) of **3j**

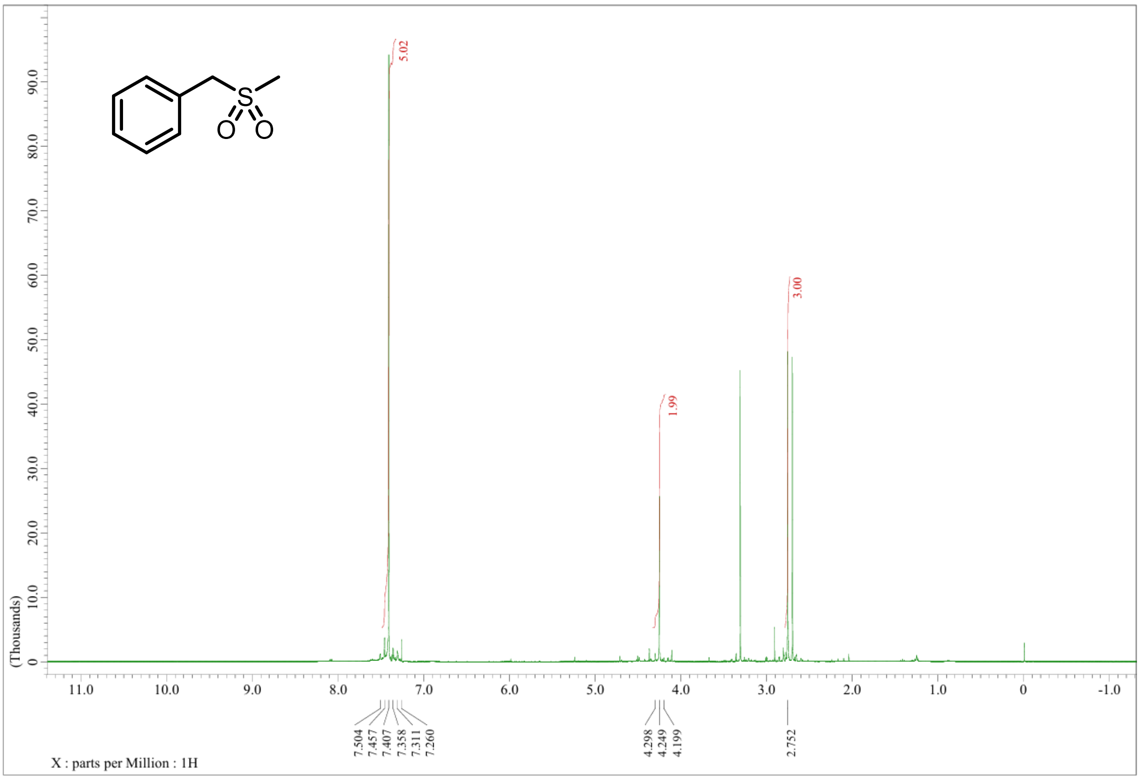

^{13}C NMR (100 MHz, $CDCl_3$, 25 °C) of **3j**

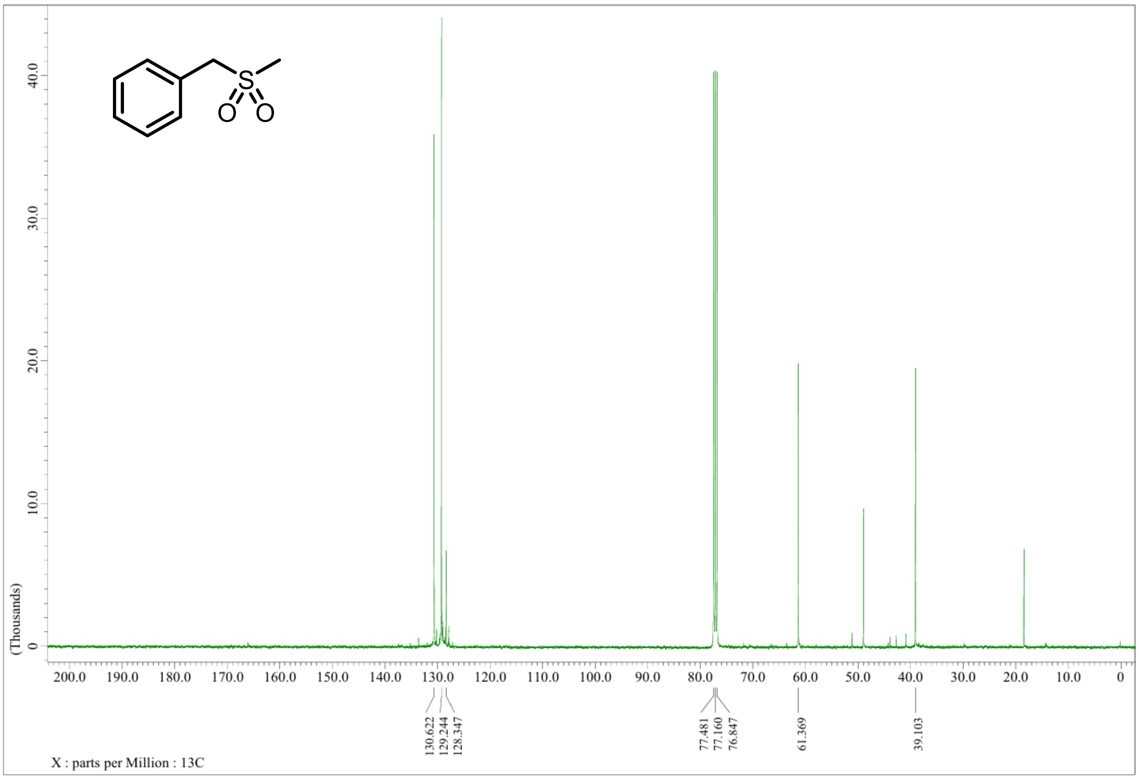

^{1}H NMR (400 MHz, $CDCl_3$, 25 °C) of **3k**

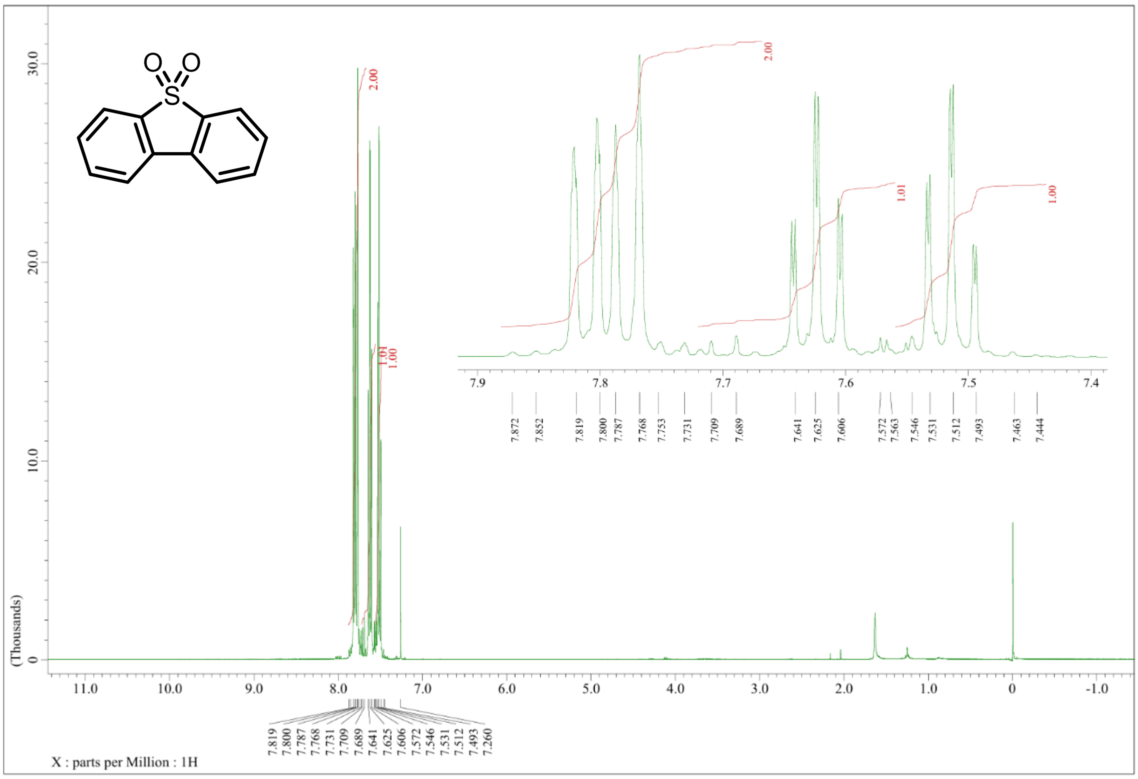

^{13}C NMR (100 MHz, $CDCl_3$, 25 °C) of **3k**

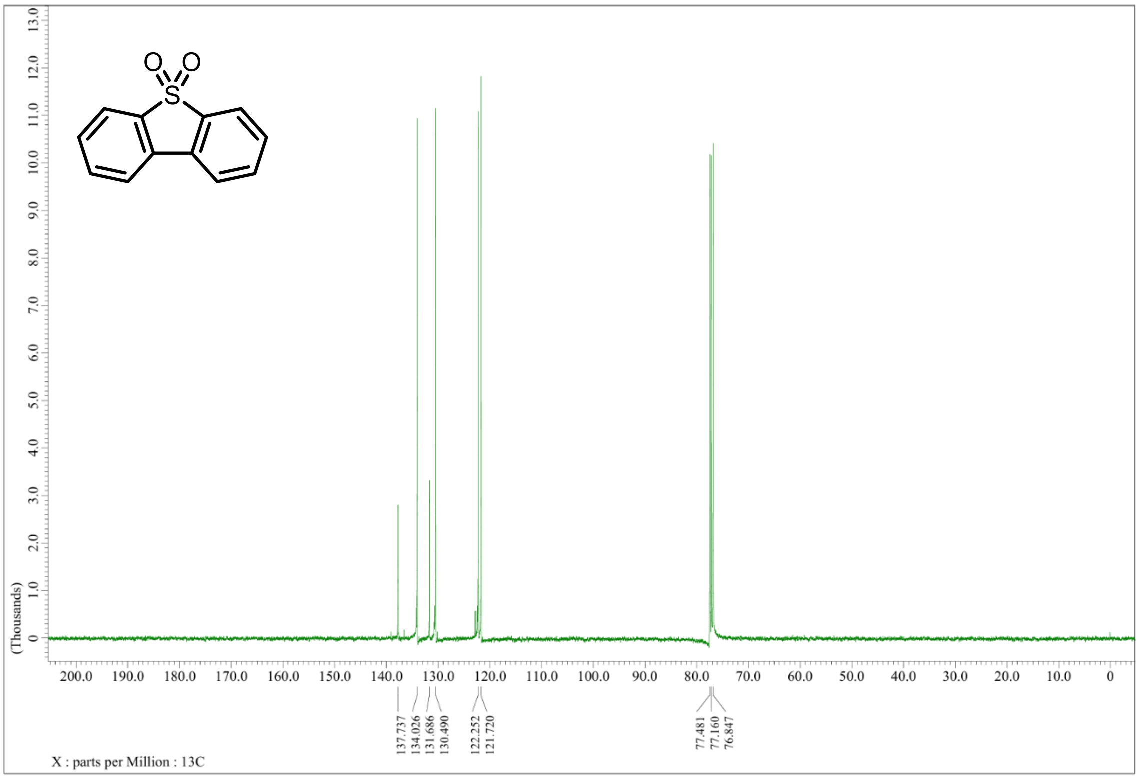

^{1}H NMR (400 MHz, $CDCl_3$, 25 °C) of **3l**

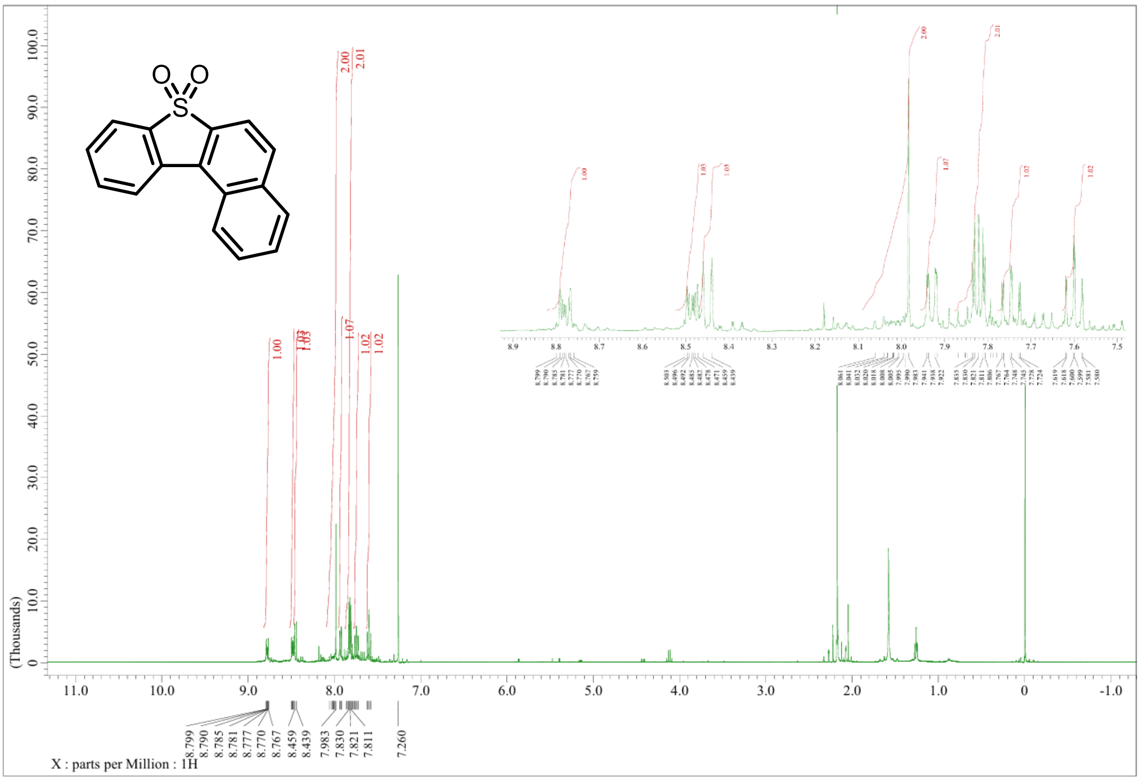

^{1}H NMR (400 MHz, $CDCl_3$, 25 °C) of **3m**

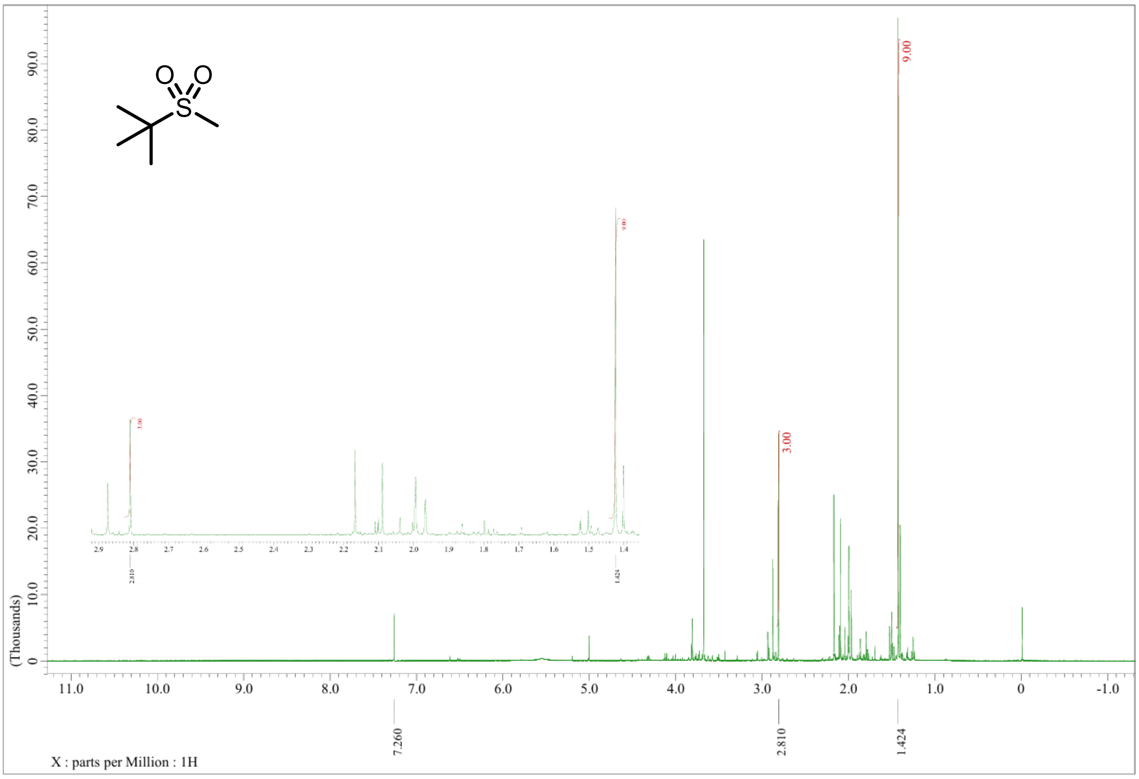

^{1}H NMR (400 MHz, $CDCl_3$, 25 °C) of **3n**

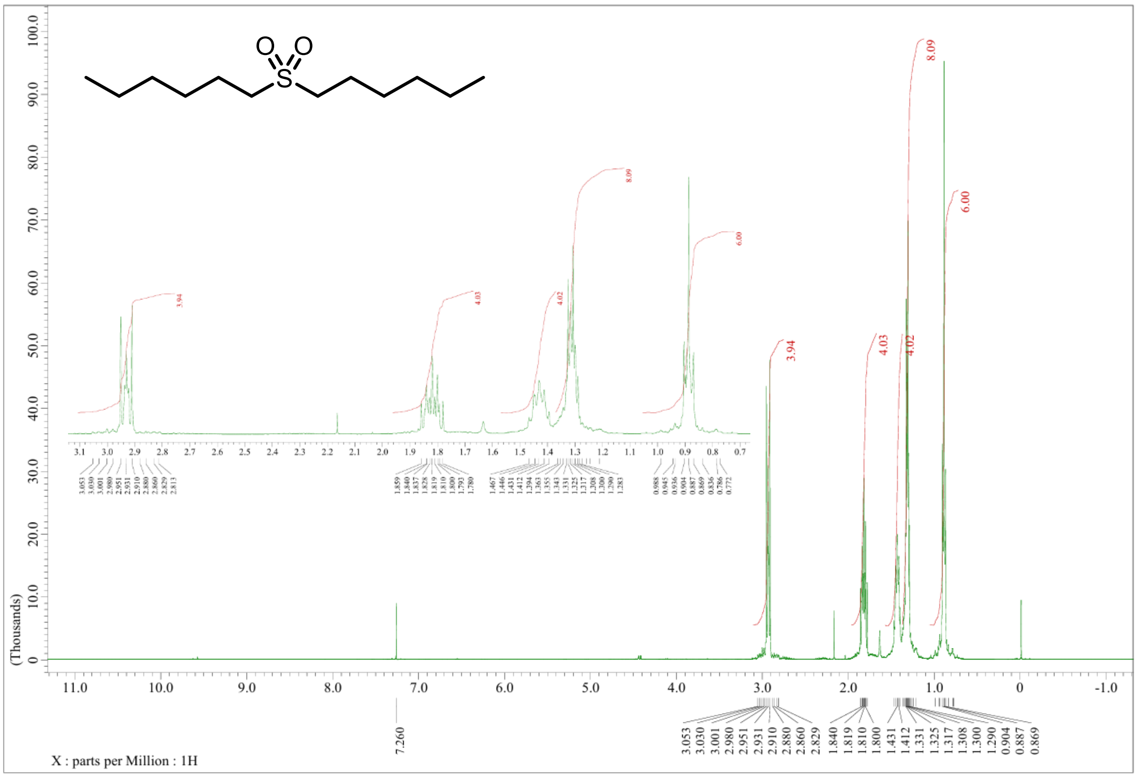

^{13}C NMR (100 MHz, $CDCl_3$, 25 °C) of **3n**

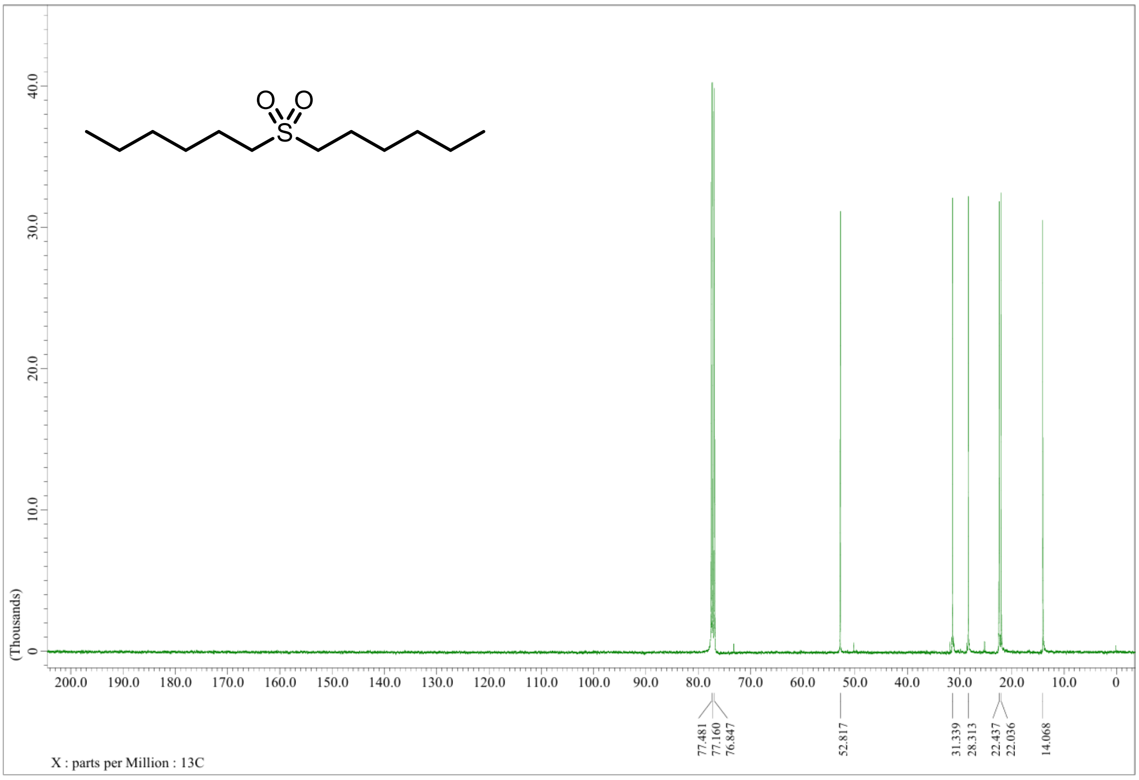

^{1}H NMR (400 MHz, DMSO-d_6, 25 °C) of **3o**

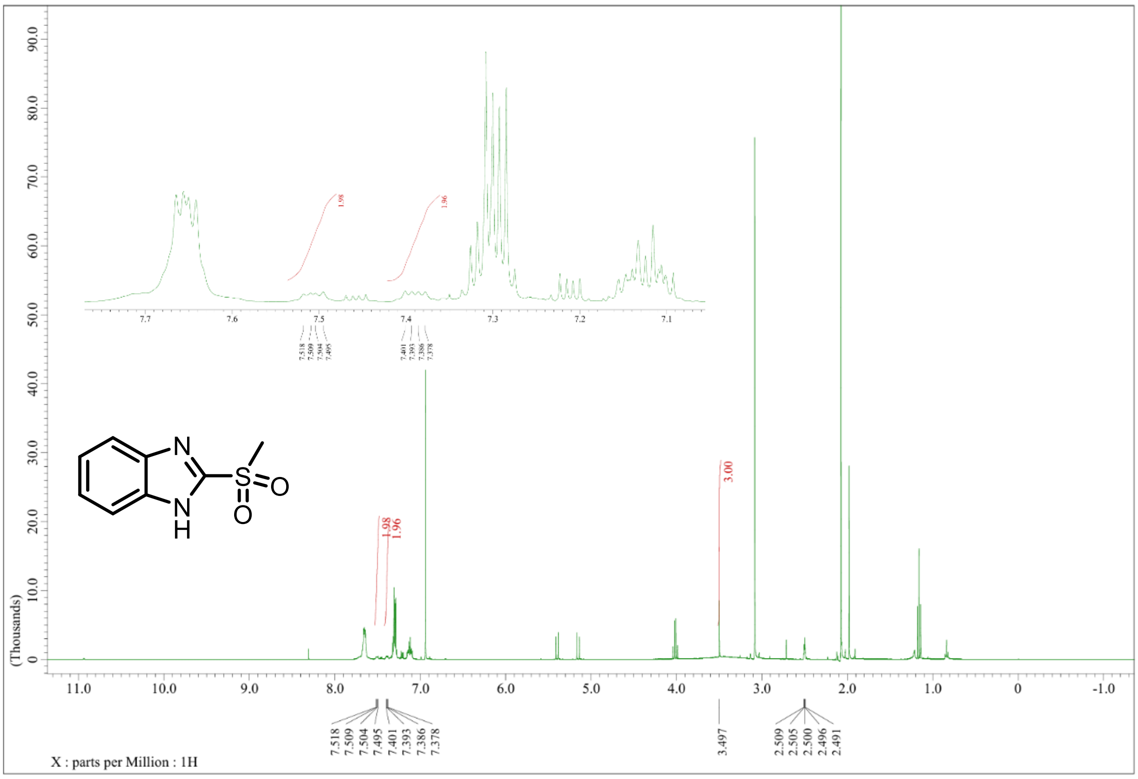

^{1}H NMR (400 MHz, D_2O, 25 °C) of **3p**

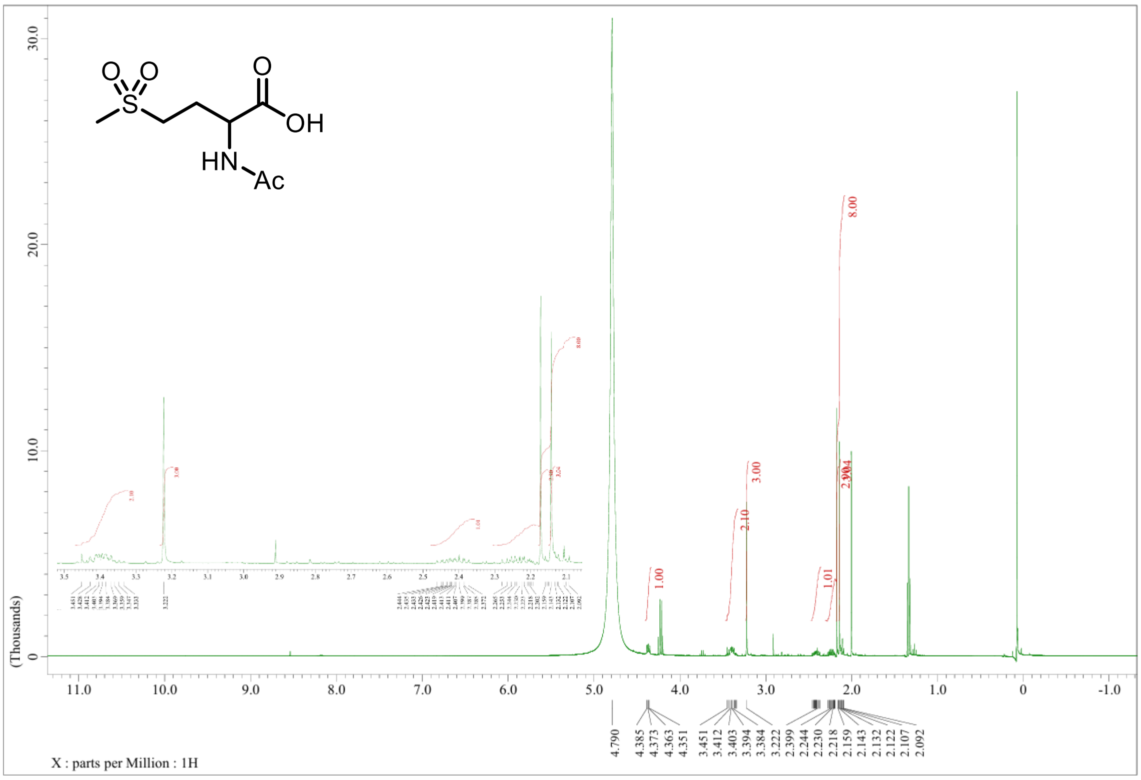

^{13}C NMR (100 MHz, DMSO-d_6, 25 °C) of **3p**

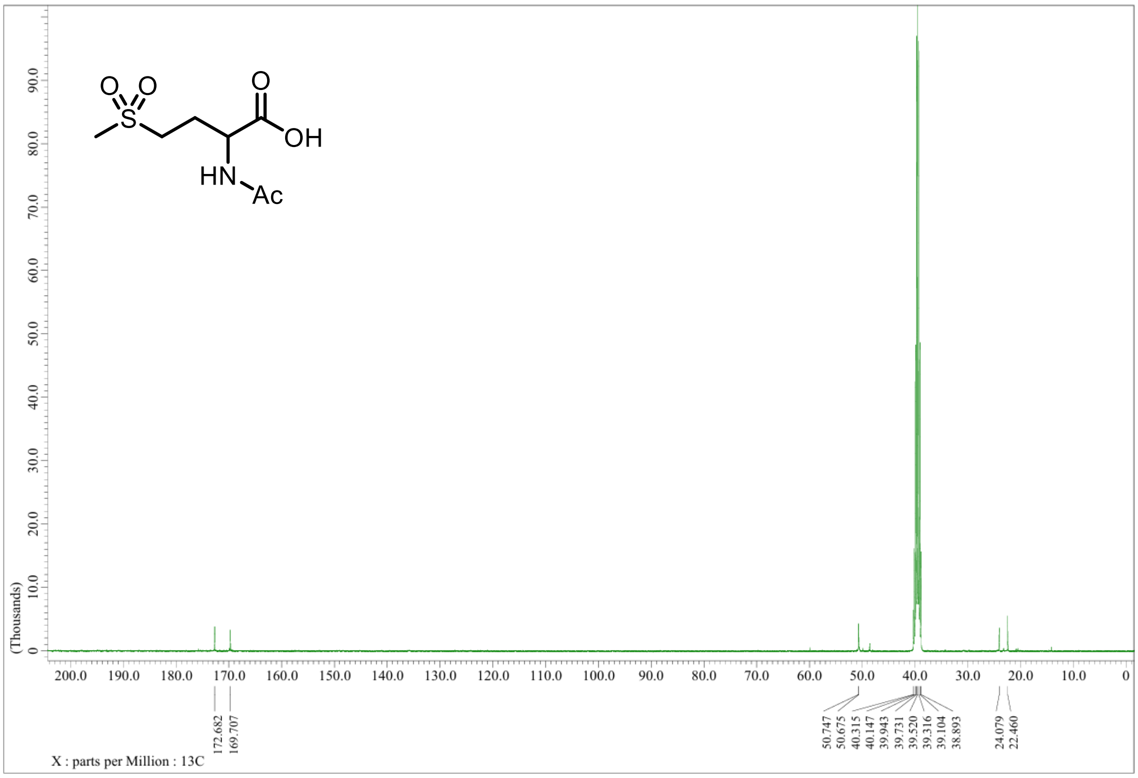

Supplement: Supplementary file 1 [file molecules-30-01912-s001.zip › molecules-3530842-supplementary.pdf]
